# DNA‑Directed Assembly of Photonic Nanomaterials for Diagnostic and Therapeutic Applications

**DOI:** 10.1002/adma.202500086

**Published:** 2025-03-19

**Authors:** Longjiang Ding, Bing Liu, Andreas Peil, Sisi Fan, Jie Chao, Na Liu

**Affiliations:** ^1^ 2nd Physics Institute University of Stuttgart Pfaffenwaldring 57 70569 Stuttgart Germany; ^2^ Max Planck Institute for Solid State Research Heisenbergstraße 1 70569 Stuttgart Germany; ^3^ State Key Laboratory for Organic Electronics and Information Displays & Jiangsu Key Laboratory for Biosensors Institute of Advanced Materials (IAM) Jiangsu National Synergistic Innovation Center for Advanced Materials (SICAM) Nanjing University of Posts and Telecommunications Nanjing 210023 China; ^4^ School of Medicine Nanjing University of Chinese Medicine Nanjing 210023 China

**Keywords:** diagnostics, DNA nanotechnology, healthcare, photonics, therapy

## Abstract

DNA‐directed assembly has emerged as a versatile and powerful approach for constructing complex structured materials. By leveraging the programmability of DNA nanotechnology, highly organized photonic systems can be developed to optimize light‐matter interactions for improved diagnostics and therapeutic outcomes. These systems enable precise spatial arrangement of photonic components, minimizing material usage, and simplifying fabrication processes. DNA nanostructures, such as DNA origami, provide a robust platform for building multifunctional photonic devices with tailored optical properties. This review highlights recent progress in DNA‐directed assembly of photonic nanomaterials, focusing on their applications in diagnostics and therapeutics. It provides an overview of the latest advancements in the field, discussing the principles of DNA‐directed assembly, strategies for functionalizing photonic building blocks, innovations in assembly design, and the resulting optical effects that drive these developments. The review also explores how these photonic architectures contribute to diagnostic and therapeutic applications, emphasizing their potential to create efficient and effective photonic systems tailored to specific healthcare needs.

## Graphical abstract

DNA‐directed assembly offers a powerful strategy for constructing structured photonic nanomaterials with precise spatial control. This review provides a comprehensive overview of recent advancements in DNA‐assembled photonic nanomaterials for diagnostics and therapeutics, highlighting key design principles, functionalization strategies, and optical effects that enhance their performance in biomedical applications.

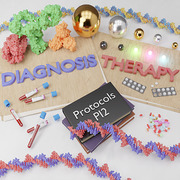

## 1 Introduction

Diagnostic and therapeutic applications are at the forefront of modern biomedicine, providing the foundation for early disease detection and effective treatment strategies. Photonic nanomaterials play an increasingly important role in these applications, leveraging their ability to precisely control and manipulate light at the nanoscale to enhance diagnostic sensitivity and therapeutic efficacy.^[^ ^1^, ^2^, ^3^, ^4^, ^5^ ^]^ This capability enables innovations, such as real‐time biosensing, high‐resolution imaging, and targeted light‐based treatments, all of which are essential for addressing current biomedical challenges. Nature offers abundant inspiration for the assembly of nanoscopic building blocks into highly ordered structures, demonstrating how geometric arrangements and chemical anisotropy influence morphology and collective properties. This principle has been instrumental in guiding the bottom‐up construction of photonic nanomaterials,^[^ ^6^ ^]^ where precise spatial arrangements of components, such as fluorophores, metal‐based nanoparticles, and quantum dots (QDs), are crucial for enhancing light‐matter interactions and tailoring optical properties. These engineered configurations provide the foundation for advanced nanophotonic systems that enable novel diagnostic tools and therapeutic modalities.

Achieving such precision in the design and fabrication of photonic nanomaterials relies on leveraging nanoscale components to develop specific optical characteristics, such as designated photonic bandgaps, enhanced light‐matter interactions, and controlled light propagation.^[^ ^4^, ^7^, ^8^, ^9^, ^10^, ^11^ ^]^ The integration of DNA nanotechnology into this process has revolutionized the assembly of photonic nanomaterials, offering unmatched precision in the spatial organization of nanoscale components.^[^ ^12^, ^13^, ^14^ ^]^ DNA, traditionally known as the medium for genetic information storage, has been redefined as a versatile structural material for nanotechnology.^[^ ^15^ ^]^ Its unique programmability and structural flexibility allow for the creation of synthetic nanostructures with remarkable precision and complexity. The programmable hybridization of DNA strands enables highly oriented bonding by employing particles or crossover‐based DNA structures as rigid templates.^[^ ^16^, ^17^, ^18^ ^]^ For instance, colloidal particles^[^ ^19^ ^]^ and DNA origami nanostructures^[^ ^20^ ^]^ with precisely positioned DNA binding sites in a tetrahedral geometry have been demonstrated to self‐assemble into diamond cubic photonic crystals. These highly ordered assemblies are crucial for optimizing light manipulation, enabling the creation of photonic systems with well‐defined 3D conformations and tailored optical properties. While DNA itself lacks intrinsic optical functionality, its ability to guide the assembly of diverse photonic building blocks has led to significant advancements in the design of photonic nanomaterials with enhanced sensitivity and specificity.^[^ ^13^ ^]^ These advancements have extended the scope of applications from cutting‐edge biosensors to targeted therapeutic platforms, such as photodynamic therapy and drug delivery systems.^[^ ^21^, ^22^, ^23^, ^24^, ^25^, ^26^, ^27^, ^28^, ^29^, ^30^, ^31^, ^32^ ^]^

This paper provides an in‐depth review of the latest advancements in DNA‐directed assembly of photonic nanomaterials, framed within the dual context of materials science and their applications in diagnostics and therapeutics. It systematically examines the fundamental principles of DNA‐directed assembly, the functionalization strategies, and the innovative structural designs that enable specific optical properties. Furthermore, the review explores how these DNA‐assembled photonic architectures contribute to diagnostic and therapeutic applications. By elucidating the synergy between DNA nanotechnology and photonics, this review underscores the transformative potential of this interdisciplinary approach. The convergence of these fields not only advances the development of next‐generation diagnostic tools and therapeutic strategies, but also establishes a versatile platform for exploring new frontiers in nanomedicine.

## 2 Brief Overview of DNA‐Directed Assembly

**Figure 1:**
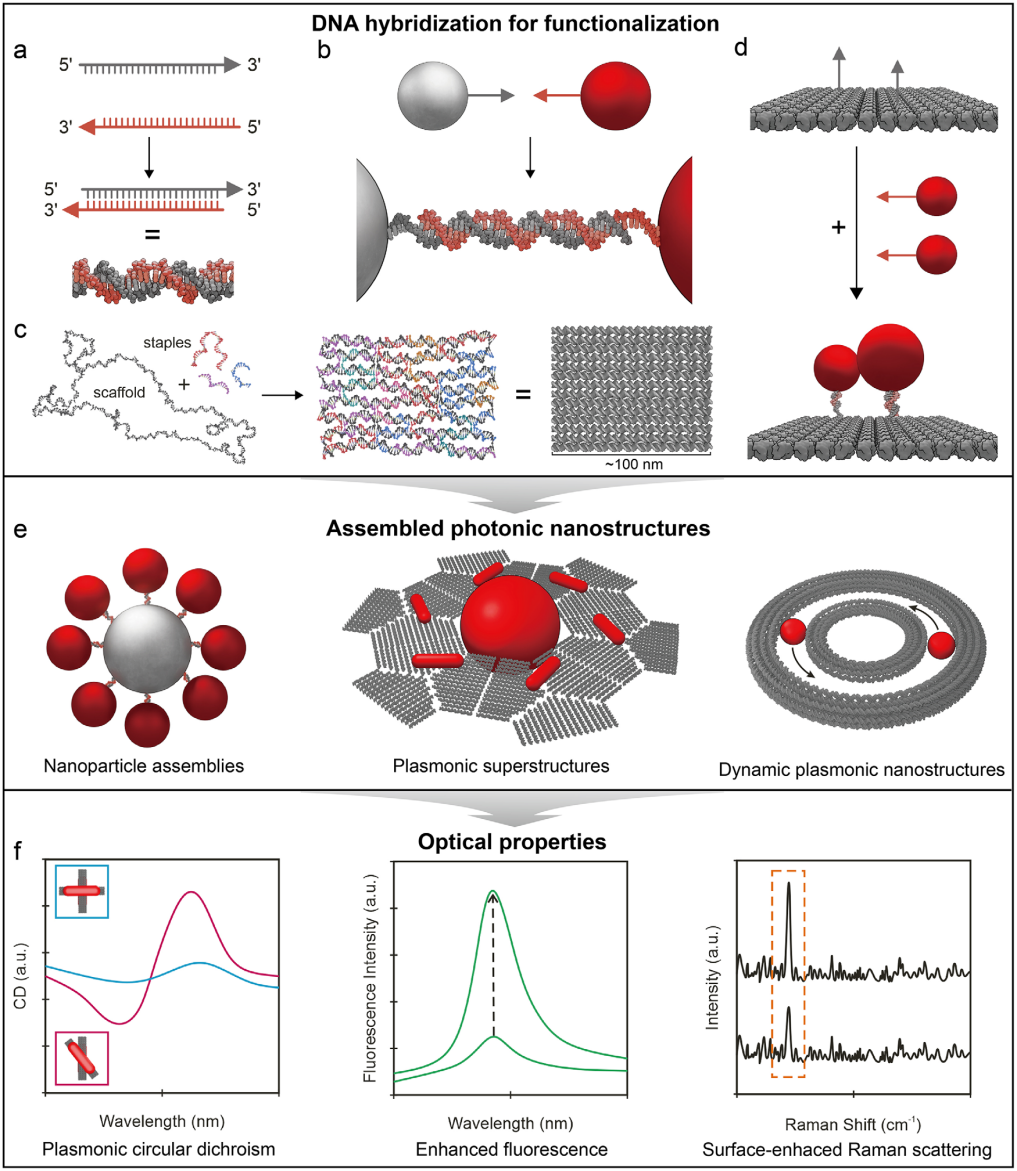
Schematic of key concepts in DNA‑directed assembly of photonic nanomaterials. a) DNA hybridization: complementary strands bind to form double‐stranded DNA (dsDNA). b) DNA hybridization drives effective interactions between DNA‐functionalized nanoparticles. c) Principle of DNA origami: scaffold and staple strands self‐assemble into predetermined shapes. d) DNA origami‐templated nanoparticle patterning. e) Assembled photonic nanostructures, including nanoparticle assemblies, plasmonic superstructures, and dynamic plasmonic structures. f) Emerging optical properties, such as plasmonic circular dichroism, enhanced fluorescence, and surface‐enhanced Raman scattering.

Although there are notable instances of self‐assembly in peptides, colloids, and polymers, synthetic assemblies often appear rudimentary compared to their natural counterparts. In biological systems, structures such as proteins, enzymes, protein cages, and viruses assemble through energy‐minimizing processes under physiological conditions. For synthetic systems, on the other hand, it is often difficult to replicate the precise targeting and control inherent to biological assemblies, due to incomplete understanding and limitations in engineering the interactions governing self‐organization. DNA stands out as a notable exception. While its primary role is genetic information storage, DNA is distinguished by its iconic double‐helical structure (**Figure** **1**a) and has been repurposed as a versatile building block for creating highly ordered, synthetic nanostructures. DNA offers unique advantages, including predictable Watson‐Crick base‐pairing, programmable sequences, tunable lengths, and unparalleled precision in designing specific interactions. Unlike natural DNA, synthetic DNA sequences are engineered not to encode proteins, but to define interaction strengths and binding specificities for self‐assembly. By applying thermodynamic principles of nucleic acid hybridization, these interactions can be engineered to achieve precise assembly outcomes, for example, driving the assembly between DNA‐functionalized nanoparticles (Figure 1b).

Intricate 3D nanostructures, such as DNA origami structures (Figure 1c), are assembled using long single‐stranded DNA molecules (“scaffold”) folded through the help of shorter synthetic DNA strands (“staples”). Although these assembled structures have no direct natural counterparts, their basic structural element, the double helix, remains identical to the DNA of our genetic code. Advances in chemical and enzymatic synthesis now allow the production of DNA molecules with customized sequences and the introduction of various chemical linkers (e.g., thiol, amino, biotin, azide), further expanding the precision and versatility of DNA self‐assembly. One prominent approach employs the synergistic hybridization of multiple DNA strands to create highly oriented bonding, often using crossover‐based DNA origami as rigid templates to guide assembly (Figure 1d).

When applied to photonic nanomaterials, DNA‐directed assembly serves as an unprecedented platform for constructing nanoscale architectures with precise spatial control (Figure 1e). Photonic building blocks, such as fluorophores, gold nanoparticles (AuNPs), and QDs, can be functionalized with DNA to enable highly specific and programmable interactions. Through DNA hybridization, these photonic elements can be organized into well‐defined chains, periodic patterns, or complex 3D superstructures with nanoscale precision. Furthermore, DNA‐directed assemblies offer dynamic adaptability, allowing structural reconfiguration in response to environmental factors, including pH, temperature, or the presence of DNA strands. The tailored optical properties of photonic nanomaterials (Figure 1f), such as plasmonic circular dichroism (CD),^[^ ^33^, ^34^ ^]^ enhanced fluorescence,^[^ ^35^, ^36^ ^]^ and surface‐enhanced Raman scattering (SERS),^[^ ^5^, ^37^, ^38^ ^]^ make DNA‐directed assembly an attractive approach for developing responsive photonic systems, which hold great potential for applications in tunable photonic devices, sensors, and advanced therapeutic materials.

One example of how DNA‐directed assemblies exploit nanoscale photophysical processes is through CD. The CD signal of DNA, observed in the UV region (220–300 nm), reflects the DNA conformation and chirality.^[^ ^39^ ^]^ DNA‐assembled chiral nanostructures can modify the CD signal, enhancing its intensity and producing new chiroptical responses in the visible and infrared ranges.^[^ ^40^ ^]^ These engineered nanostructures enable unique phenomena, such as modulating CD signals, which are difficult to achieve using traditional methods. The ability to fine‐tune the CD response via DNA origami highlights the potential of DNA‐directed approaches for manipulating light‐matter interactions at the nanoscale, providing valuable insights into their structural and chiroptical properties.

## 3 DNA‐Functionalized Photonic Building Blocks

Imparting DNA bonding capability to photonic building blocks is crucial for DNA‐directed construction of photonic materials. For fluorophores, several key methodologies are employed.^[^ ^41^ ^]^ Non‐covalent attachment that relies on electrostatic interactions, hydrophobic interactions, or affinity‐based schemes (e.g., biotin‐streptavidin) is generally simple to implement. In contrast, covalent conjugation is one of the most effective approaches, enabling stable attachment of DNA strands to fluorophores using chemical linkers such as amine‐reactive or thiol‐reactive chemistries. They often exhibit enhanced stability compared to noncovalent approaches. In addition, dibenzylcyclooctyne(DBCO)‐azide click chemistry, offers a highly efficient and specific means of attachment, allowing for precise bonding with minimal disruption to the photonic properties of the fluorophores. Such precision is critical for manipulating the optical integrity of the fluorophores during assembly. Notably, researchers can obtain DNA molecules conjugated with various fluorophores from commercial suppliers, greatly simplifying experimental workflows.

For nanoparticles, two primary strategies are employed to functionalize their surfaces with DNA: uniform and anisotropic modifications. Uniform modification ensures consistent DNA attachment across the entire nanoparticle surface, enabling predictable interactions and isotropic assembly. In contrast, anisotropic modification selectively targets specific regions of the nanoparticle surface, allowing for the construction of complex, oriented structures with precise control over spatial arrangements and interactions. This regiospecific approach is particularly valuable for advanced photonic devices and therapeutic systems, where spatial arrangement directly influences functionality, such as in plasmonic hotspots or directed energy transfer. Together, uniform and anisotropic DNA modifications serve complementary purposes, optimizing the performance and applicability of photonic nanomaterials. By choosing the appropriate functionalization strategy, researchers can tailor DNA‐directed assembly to meet the specific requirements of applications in diagnostics, sensing, and therapeutic platforms.

### 3.1 Uniform Modification of Nanoparticles with DNA

**Figure 2:**
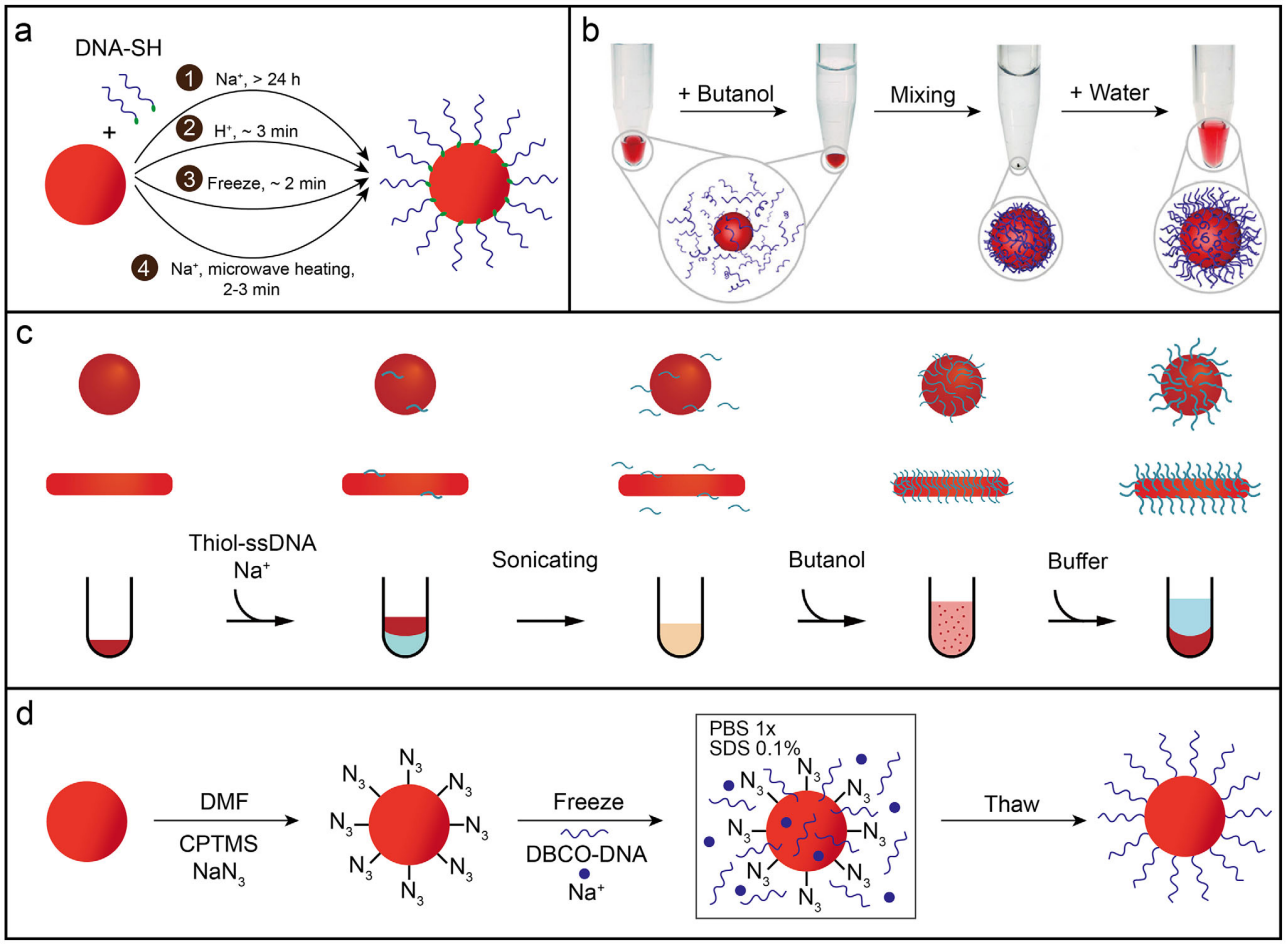
Approaches for uniform modification of nanoparticles with DNA. a) Scheme for binding DNA to AuNPs by (1) salt‐aging, (2) lowering pH, (3) freezing, and (4) microwave heating. b) INDEBT process for the flash synthesis of DNA‐coated AuNPs. Reproduced with permission.^[^ ^52^ ^]^ Copyright 2021, American Chemical Society. c) Diagram illustrating an ultrafast approach to directly prepare high‐density DNA‐conjugated QDs and QRs from an organic solution through a dehydration and rehydration method. Reproduced with permission.^[^ ^54^ ^]^ Copyright 2023, AAAS. d) Click‐Chemistry approach for DNA functionalization of diverse nanoparticles.^[^ ^56^ ^]^

The modification of nanoparticles with DNA is typically achieved through the covalent or non‐covalent attachment of DNA strands to their surfaces, a concept originating from two seminal papers published back‐to‐back in Nature in 1996.^[^ ^42^, ^43^ ^]^ The uniform modification of metal nanoparticles with DNA was first demonstrated by Mirkin and colleagues,^[^ ^42^ ^]^ who employed Au‐S chemistry to evenly attach multiple DNA strands to spherical AuNPs. This method overcame the inherent charge repulsion between negatively charged DNA molecules and citrate‐stabilized, negatively charged AuNPs by introducing the salt‐aging method (**Figure** **2**a). In this process, salt was gradually added to a mixture of thiol (SH)‐^[^ ^44^ ^]^ or poly(A)‐tagged^[^ ^45^ ^]^ DNA and AuNPs, mitigating charge repulsion and facilitating attachment. Using the salt‐aging approach, Salaita and colleagues demonstrated that spatially patterned oligonucleotides on nanoparticle surfaces could dramatically improve binding affinity. Specifically, nanoparticles with heteromultivalent surface patterns showed a ≈23‐fold higher affinity compared to chemically identical, randomly patterned nanoparticles.^[^ ^46^ ^]^ However, this method requires over 2 days to complete, making it time‐consuming. To accelerate the process, alternative strategies have been introduced, including the incorporation of acids,^[^ ^47^ ^]^ surfactants,^[^ ^48^ ^]^ or polymers,^[^ ^49^ ^]^ although these additional reagents may limit the broader applicability of the modified nanoparticles.

Another reagent‐free freeze‐thaw method was then developed to efficiently attach SH‐DNA to AuNPs ranging from 5 nm to 100 nm in diameter, achieving DNA densities 20–30% higher than those obtained via conventional salt‐aging.^[^ ^50^ ^]^ Interestingly, even higher DNA densities could be achieved simply by freezing the mixture. The authors hypothesized that the crystallization of water during freezing might push DNA, AuNPs, and salt away from the growing ice crystals, leading to a high local concentration of these components and enhanced DNA attachment kinetics. Recently, a microwave‐assisted heating‐dry method was developed to concentrate DNA, AuNPs, and salt.^[^ ^51^ ^]^ In this process, a commercial microwave oven was used to quickly evaporate water, completing the labeling within minutes without the need for additional reagents. Remarkably, this method could also be used with non‐thiolated DNA by employing poly(T) tags of five bases in length. Conversely, it is not universally applicable to all non‐thiolated DNA sequences, as only those containing a minimum of five consecutive T bases can be utilized, which may limit its potential applications.

Beyond these salt‐based physical acceleration methods, Deng and colleagues developed a chemical strategy to enhance the attachment of SH‐DNA to AuNP surfaces without the necessity of salt.^[^ ^52^ ^]^ This approach, called instant dehydration in butanol (INDEBT), was inspired by the common laboratory practice of using butanol extraction to remove staining dyes and excess water from DNA samples. The authors observed that continued butanol additions eventually caused the water phase to disappear, leaving behind segregated DNA and salts. Based on this, they devised an INDEBT technique for rapid DNA modification, leveraging the concept of a highly concentrated, dehydrated, and uniformly mixed “solid solution” of AuNPs and DNA. Typically, this INDEBT‐based method involves two swift solution‐mixing steps (Figure 2b). In the first step, an aqueous solution of SH‐DNA and AuNPs was introduced into a butanol phase. The butanol volume was carefully adjusted to ensure the complete removal of water, which facilitated the rapid conjugation of DNA. In the second step, a fresh water phase was added to rehydrate and recover the DNA‐coated AuNPs that formed. Each step took only a few seconds. In addition to chemical strategies, an acoustic levitation device has recently been developed to synergistically drive DNA conjugation by combining dehydration and ligand shedding.^[^ ^53^ ^]^ This technique eliminates the need for additional chemicals and introduces a novel mechanism of DNA grafting via acoustic waves, enabling the reconfiguration of ligands on nanoparticle surfaces. While it facilitates the universal modification of AuNPs with an ultra‐high density of DNA strands, the widespread adoption of this method will depend significantly on the availability of this custom‐built device or the development of commercially viable alternatives.

Apart from AuNPs and other noble metal‐based nanoparticles, an ultrafast dehydration‐rehydration method has been demonstrated for preparing high‐density DNA‐conjugated QDs and quantum rods (QRs) directly from organic solutions (Figure 2c).^[^ ^54^, ^55^ ^]^ In this method, QDs/QRs, commercially available and dispersed in organic solvents, were combined with an aqueous solution containing excess SH‐DNA and sodium salt, resulting in the formation of a liquid double layer. Brief sonication created an emulsion, allowing some QDs/QRs to transfer to the aqueous phase through partial ligand exchange. Adding butanol facilitated further dehydration, condensing DNA onto the QDs/QRs and enabling efficient conjugation. This state resembled the “solid solution” described in the INDEBT technique for AuNPs and DNA. The DNA‐functionalized QDs/QRs were subsequently recovered by adding aqueous buffer or pelleting and removing the butanol. This method is applicable to QDs/QRs of varying sizes, aspect ratios, and shell compositions, and is also compatible with various organic solvents commonly used for QDs/QRs, such as chloroform, toluene, or hexane. The resulting DNA‐functionalized QDs/QRs exhibit high DNA density, excellent stability, and strong binding affinity in various salted aqueous buffers.

To expand the type of nanoparticles that can be functionalized with DNA, a freeze‐thaw method combined with DBCO‐azide click chemistry was recently developed, enabling conjugation of DNA to oxide nanoparticles, polymer‐based particles, and metallic nanostructures.^[^ ^56^ ^]^ For example, silica and silicon nanoparticles (Figure 2d) can be functionalized by first treating them with chloride‐terminated silane (CPTMS) in anhydrous dimethylformamide (DMF), followed by the addition of sodium azide (NaN₃). DBCO‐decorated DNA is then attached to the azide‐functionalized nanoparticles through stable triazole bond formation. To expedite this process, the suspension of azide‐functionalized nanoparticles and DBCO‐DNA in phosphate‐buffered saline (PBS) is frozen at −20 °C for 2 h, which increases DNA concentration at the nanoparticle surface. After thawing, the suspension is briefly sonicated, yielding colloids with high DNA surface densities of ≈0.2 molecules nm^−^^2^. This approach complements traditional thiol‐based functionalization techniques, considerably broadening the repertoire of DNA‐functionalized nanoparticles available for various applications. Given the high yield of the click‐chemistry reaction, the quality of azide group modification determines DNA functionalization efficiency, making the surface modification of azide groups on nanoparticles, especially those of different materials, critical for optimal results.

### 3.2 Anisotropic Surface Functionalization of Nanoparticles with DNA

The anisotropic surface modification of nanoparticles with DNA, in contrast to uniform DNA functionalization, was first introduced by Alivisatos and colleagues.^[^ ^43^ ^]^ They demonstrated that small AuNPs (1–2 nm) could be modified with just a single DNA strand of specific length and sequence per particle, enabling the assembly of discrete “nanocrystal molecules” instead of bulk structures. Since then, various DNA‐directed anisotropic surface encoding approaches have been developed and applied to different nanoparticles.

**Figure 3:**
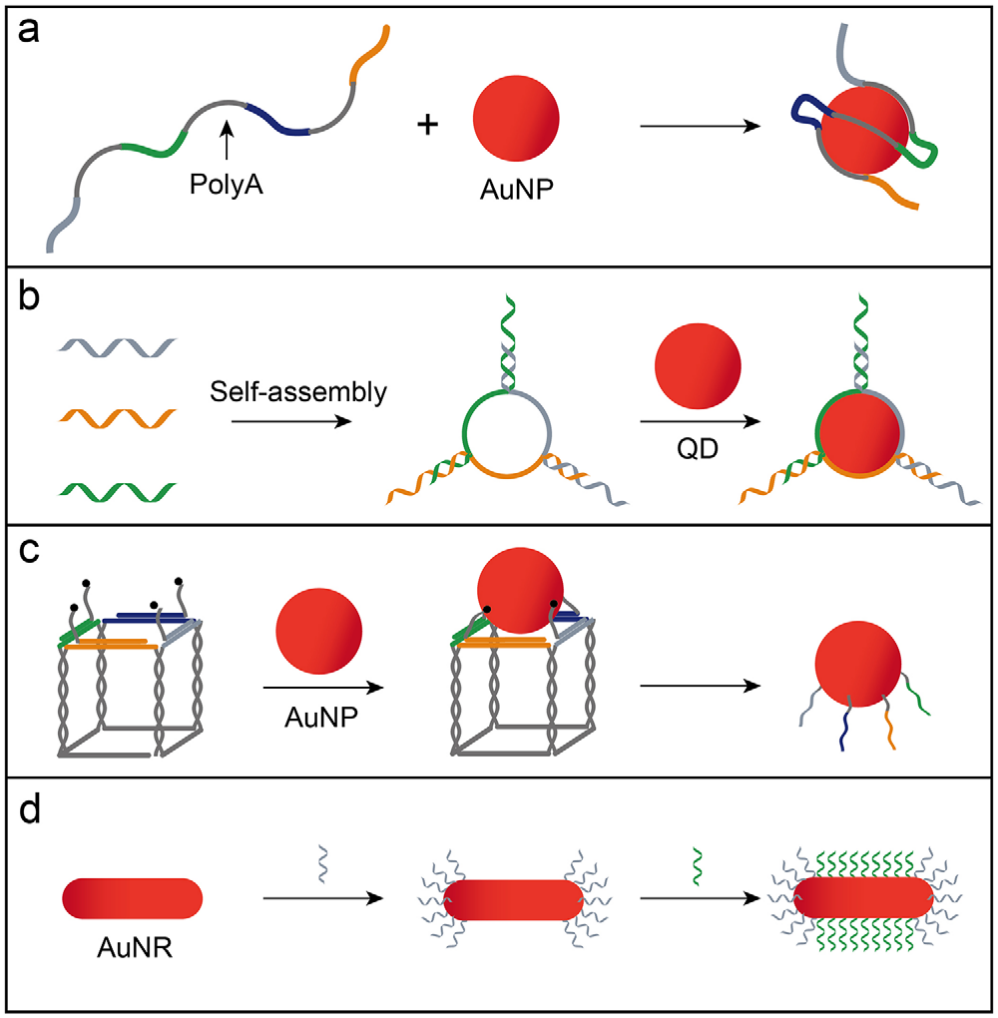
Approaches for anisotropic modification of nanoparticles with DNA. a) Schematic of ssDNA‐functionalized AuNPs, where the DNA comprises alternating polyA and non‐polyA domains. b) Schematic of valence‐modified quantum dots assembled with multi‐armed DNA frameworks. c) DNA printing strategy, which utilizes DNA nanostructures as templates to organize bonding sites into predefined patterns and subsequently transfer these patterns onto isotropic AuNPs. d) Shape‐dependent modification of anisotropic AuNRs using distinct DNA sequences.

Building on Alivisatos’ concept of DNA “codons”, controlling the number of DNA strands attached to each nanoparticle has become one of the most straightforward approaches to achieving anisotropic surface functionalization. However, direct attachment of single‐stranded DNA (ssDNA) to nanoparticles often results in products with valences following a Poisson distribution, leading to a mixture of desired valences, unconjugated particles, and multivalent byproducts.^[^ ^57^ ^]^ To address this, steric hindrance strategies have been successfully employed to produce monovalent AuNPs^[^ ^58^ ^]^ and QDs,^[^ ^59^ ^]^ enhancing the yield of nanoparticles modified with a single DNA strand. When ssDNA is designed with alternating sticky polyA domains and non‐sticky bonding domains, the resulting nanoparticles can exhibit valence‐bond analogs (**Figure** **3**a).^[^ ^60^ ^]^ PolyA readily adsorbs onto AuNP surfaces, with its bonding regions extending into the surrounding solution, allowing for selective and reversible hybridization interactions. By carefully designing the sequence, length, and arrangement of these regions, a variety of programmable atomic equivalents can be created with highly controlled valence. However, when using this approach for multivalent encoding, the ssDNA encoder must be relatively long, and currently, only tetravalent modifications have been reported, encoded by seven domains. Additionally, structured DNA scaffolds can substitute for ssDNA, allowing the creation of multivalent products. For example, a DNA‐programmed strategy combining steric exclusion with electrostatic repulsion has enabled high‐yield, modular valence engineering of QDs.^[^ ^61^ ^]^ As shown in Figure 3b, neutral mPEG reduced surface charge to facilitate DNA attachment, while appropriately sized chimeric DNA wrapped around the QDs, preventing further binding.

To improve spatial addressability, transferring defined DNA patterns from templates to nanoparticles has proven to be an effective approach.^[^ ^62^ ^]^ Sleiman and colleagues pioneered the molecular printing of DNA strands from reusable DNA nanoscaffold templates.^[^ ^63^ ^]^ As illustrated in Figure 3c, a maximum of four unique DNA strands on the surface of a cubic DNA template and subsequently transferred to a AuNP in one cycle. When the DNA cube underwent denaturation, the patterned DNA strands were transferred onto the nanoparticle surface, maintaining their spatial configuration and molecular recognition characteristics. Similarly, exogenous printing processes within DNA icosahedron cages have been employed for 3D molecular printing on AuNPs.^[^ ^64^ ^]^ Although the assembly and purification of DNA icosahedron cages can be complex and time‐consuming, this approach enables simultaneous control over both the number and spatial arrangement of DNA strands on the surface of AuNPs, allowing for tunable regulation from a single strand to multiple strands and from 1D to 3D positioning. Furthermore, DNA origami structures provide sturdy and expansive frameworks for transferring DNA patterns. Staple strands in the DNA origami serve as recognition sites for anchoring nanoparticles with high precision and addressability. A DNA origami‐based nanoimprinting lithography technique was developed for transferring DNA patterns from 2D DNA origami onto AuNPs,^[^ ^65^ ^]^ gold nanorods (AuNRs)^[^ ^66^ ^]^ and gold nanocubes (AuNCs).^[^ ^67^ ^]^ Rather than breaking the DNA structures, these methods employ strand‐displacement reactions to detach nanoparticles from the parent origami templates, allowing for intact pattern transfer. Additionally, 3D DNA origami frames have been used to transfer multiple types of DNA molecules onto the surface of nanoparticles, enabling precise, single‐molecule control over functionalization. This molecular stamping approach allows for the spatially defined arrangement of different molecules, facilitating the creation of monochromatic and polychromatic nanoscale patchy.^[^ ^68^ ^]^

Compared to additive strategies, the DNA origami‐based subtractive approach provides an alternative method for modulating anisotropic DNA surface modifications and enabling precise nanoparticle manipulation.^[^ ^69^, ^70^ ^]^ An enzymatic nanorobot arm anchored to a nanoparticle and selectively excised DNA, introducing anisotropic modifications. A rectangular DNA origami scaffold then guided the precise placement of these modifications, enhancing addressability and programmability.^[^ ^69^ ^]^ Inspired by macroscopic subtractive manufacturing, a DNA origami barrel‐directed subtractive patterning strategy was recently introduced to selectively deactivate DNA ligands on nanosphere surfaces, creating precise and oriented patches.^[^ ^70^ ^]^ In this approach, DNA barrels were used to partially block the surface of DNA‐coated spherical nanoparticles, leaving regioselective DNA patches made of densely packed DNA strands on the exposed regions. The number and size of the patches could be controlled by adjusting the bonding positions and height of the DNA barrels. For instance, blocking half of the nanosphere's surface with a DNA barrel resulted in the formation of a Janus nanoparticle with a single exposed DNA patch. Shielding the central region with DNA barrels while leaving the top and bottom exposed produced a symmetrical triblock Janus particle with two active patches. This approach offers the potential to achieve previously inaccessible structures, thereby advancing applications in metamaterial fabrication and optomechanical systems.

Regiospecific functionalization of anisotropic nanoparticles is easier to achieve than that of isotropic ones due to the presence of multiple localized surfaces with varying chemical or physical properties, enabling selective modification for regioselective DNA attachment. For example, AuNRs capped with a cetyltrimethylammonium bromide (CTAB) bilayer exhibits preferential binding of CTAB to the (100) side facets, leaving the (111) end facets chemically more reactive. This property enables selective functionalization of the AuNR ends with SH‐DNA, while immobilizing distinct DNA strands on the sides through a two‐step modification process (Figure 3d).^[^ ^71^ ^]^ Similar regioselective functionalization strategies have been applied to anisotropic gold nanotriangles (AuNTs),^[^ ^72^ ^]^ gold nanostars (AuNSs)^[^ ^73^ ^]^ and AuNCs.^[^ ^74^ ^]^ However, the precision of this shape‐based modification method is not exceptionally high, which may limit its applicability in certain contexts.

Given the ease of regiospecific functionalization in anisotropic nanoparticles, researchers have sought methods to impart regioselectivity to isotropic nanoparticles prior to DNA modification. In addition to physically blocking the nanoparticle surface with substrates,^[^ ^75^, ^76^ ^]^ chemical blocking strategies have also been developed. For example, a one‐step, solution‐based method was demonstrated to decorate AuNPs with DNA patches using ligand competition.^[^ ^77^ ^]^ Weizmann and colleagues controlled interfacial tension between nanoparticles, solvents, and copolymers to create nanoscale DNA patches on nanospheres, nanorods, nanoprisms, and nanocubes.^[^ ^78^ ^]^

## 4 Photonic Nanomaterials Assembled Using DNA Strands

### 4.1 Nanoparticle Assembly Based on Uniform DNA Modification

Nanoparticles can be assembled through the creation of DNA bridges, regardless of the approach used to attach DNA strands to them. These bridges are formed when a strand linked to one particle hybridizes with a strand attached to another, or when two attached strands bind to a third linking strand.^[^ ^16^ ^]^ Uniform DNA coverage enhances interparticle stability by enabling the formation of multiple DNA bridges, ensuring consistent surface coverage and symmetric interactions. This uniformity is critical for orderly assembly, which is essential for applications requiring long‐term stability and reliable optical properties.

**Figure 4:**
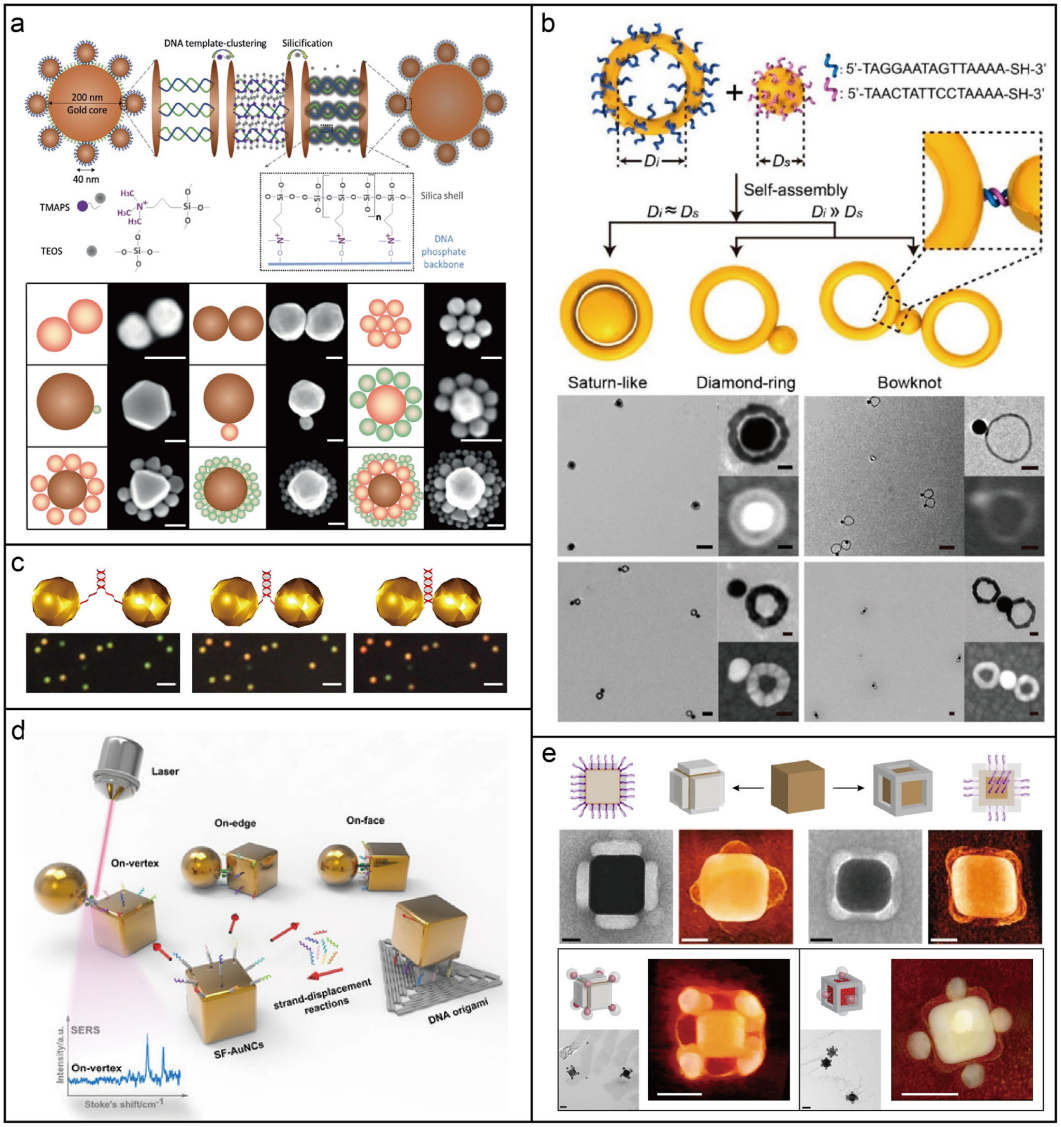
Photonic nanomaterials are assembled using DNA strands. a) Structurally resilient plasmonic nanocavities via standard DNA hybridization reactions, followed by DNA silicification.^[^ ^80^ ^]^ Copyright 2021, Wiley. b) Assembly of gold nanorings and nanospheres, directed by shape complementarity and functionalized with complementary DNA strands, into distinct hybrid plasmonic nanostructures with precisely defined morphologies.^[^ ^82^ ^]^ Copyright 2020, American Chemical Society. c) Plasmonic AuNP dimers linked by a transverse DNA double helix, with interparticle distance actively reduced by increasing local ionic strength.^[^ ^94^ ^]^ Copyright 2021, American Chemical Society. d) DNA origami‐based nanoprinting transfers predefined DNA strands onto AuNC surfaces, enabling stereo‐controlled metallic nanostructures with enhanced SERS signals via single dye molecule anchoring in hotspots.^[^ ^67^ ^]^ Copyright 2021, Wiley. e) Self‐assembled structures formed by combining regioselectively surface‐encoded patchy AuNCs and AuNPs. Reproduced with permission.^[^ ^78^ ^]^ Copyright 2019, Nature Publishing Group.

Since the initial attempt to assemble uniformly DNA‐grafted AuNPs was made in 1996,^[^ ^42^ ^]^ substantial progress has led to the development of diverse nanoparticle assemblies with interesting photonic properties and potential applications in biology.^[^ ^79^, ^80^, ^81^, ^82^, ^83^, ^84^, ^85^, ^86^, ^87^, ^88^, ^89^, ^90^, ^91^ ^]^ For example, as shown in **Figure** **4**a, plasmonic nanocavities exhibiting structural resilience were constructed by hybridizing complementary DNA strands, which were uniformly bound to the core and satellite AuNPs.^[^ ^80^ ^]^ Subsequent DNA silicification was applied to enhance structural stability, chemical inertness, and nanogap control, simultaneously generating large and precisely controlled local electromagnetic field enhancements. These silicified DNA scaffolds have proven versatile in spatially organizing plasmonic nanoparticles into hierarchical assemblies, ranging from dimers to core‐satellite‐satellite configurations. Shape complementarity has further expanded the diversity of assembled structures. As shown in Figure 4b, gold nanoring‐based heterogeneous nanostructures, including Saturn‐like, diamond‐ring, and bowknot shapes, were fabricated with high yield.^[^ ^82^ ^]^ In this approach, gold nanorings and nanospheres are uniformly functionalized with complementary ssDNA. DNA sequence recognition drives self‐assembly, while shape complementarity dictates the morphology of the assembled nanostructures.

Beyond nanoparticle oligomers, large‐scale photonic assemblies with higher‐dimensional architectures have been successfully constructed. Combining DNA‐directed assembly with top‐down lithography enables the creation of reconfigurable plasmonic nanoparticle superlattices on gold surfaces. Precise control over shape, size, and interparticle distances was achieved using electron beam lithography to fabricate 1D pores in polymer‐coated gold substrates.^[^ ^79^ ^]^ The substrate base is uniformly functionalized with ssDNA, which hybridized with complementary DNA strands to immobilize AuNPs (spheres, cubes, or disks) within the pores. Layer‐by‐layer assembly using designed DNA sequences produces oriented superlattices with two‐ and three‐layer architectures. Removing the polymer template reveals 2D arrays with adjustable interparticle distances, modulated by altering the solvent composition. These assemblies exhibit tunable optical properties, such as resonance shifts in absorption spectra. Additionally, linker DNA strands facilitate the assembly of uniformly DNA‐functionalized AuNPs into diverse 3D crystalline architectures.^[^ ^18^ ^]^ By tuning the crystal habit and internal nanoparticle arrangement, superlattices with tailored optical properties have been achieved.^[^ ^92^ ^]^ For instance, low‐symmetry kagome superlattices assembled using DNA‐coated gold bipyramids exhibit facet‐dependent lattice plasmon resonances and complex light emission properties.^[^ ^81^ ^]^

### 4.2 Nanoparticle Assembly Based on Anisotropic DNA Modification

Although uniform DNA modification enhances nanoparticle stability and binding efficiency, it generally lacks the spatial specificity required for precise, asymmetric arrangements where orientation‐dependent interactions are essential.^[^ ^93^ ^]^ Anisotropic surface functionalization addresses this limitation by enabling directional assembly. As shown in Figure 4c, plasmonic dimers can be formed by assembling polycrystalline 40 nm AuNPs with a DNA strand positioned perpendicular to the axis of the dimer.^[^ ^94^ ^]^ One AuNP is modified with a 50‐base‐long ssDNA containing a trithiol group at the 5′‐end, while the other is conjugated with its complementary strand, which carries a trithiol moiety at the 3′‐end. Hybridization drives self‐assembly and ionic strength modulation reduces the interparticle distance to sub‐2 nm gaps in 80% of samples. Incorporating ATTO647N dye molecules onto the DNA strands enables the observation of strongly coupled hybrid modes scattering spectra.

Anisotropic colloidal molecules can also be fabricated by programming the sequence, order, and length of alternating sticky poly(A) and bonding domains on DNA strands. For example, anisotropic colloidal molecules assembled using AuNPs with diameters of 5, 10, 15, and 20 nm display pronounced negative peaks in the plasmonic absorption region of the CD spectrum.^[^ ^60^ ^]^ Interparticle distance can be controlled using different configurations of DNA bonding domains, with experimentally determined averages, ranging from ≈1.7 nm for a kissing‐loop motif to ≈6.0 nm for a partially double‐stranded structure.^[^ ^95^ ^]^ Recently, chemical welding of these colloidal molecules with Ag₂S primers has been demonstrated, allowing for the formation of AgAu alloy junctions that exhibit charge transfer plasmon generation.^[^ ^96^ ^]^

In addition, anisotropic DNA‐functionalized nanoparticles can guide the crystallization of Au atoms. ssDNA anchored on gold nanoseeds can regulate gold precursor distribution gradients, controlling the morphology of the resulting crystalline nanostructures.^[^ ^97^ ^]^ These plasmonic asymmetric nanoarchitectures exhibit enhanced electromagnetic fields,^[^ ^98^ ^]^ optical energy density,^[^ ^99^ ^]^ and refractive index sensitivity.^[^ ^100^ ^]^

DNA molecular printing endows nanoparticles with nanoscale addressability similar to that of DNA nanostructures.^[^ ^62^ ^]^ DNA molecular patterns with precisely arranged spatial information on the particle surface facilitate self‐directed assembly into geometrically regulated configurations. As shown in Figure 4d, toehold‐initiated strand displacement reactions, strategically designed for precise control, facilitate the transfer of DNA strands with predefined numbers, sequences, and spatial arrangements onto the surface of AuNCs.^[^ ^67^ ^]^ These DNA strands on AuNCs enable selective binding with AuNPs, leading to the formation of stereo‐controlled AuNC‐AuNP nanostructures with well‐defined geometries and compositions. Additionally, positioning a single dye molecule within nanoscale gaps between closely spaced nanoparticles enhances the localized electromagnetic fields in hotspot regions, thereby significantly amplifying the single‐molecule SERS signal.

Patchy nanoparticles, functionalized with multiple DNA strands provide better spatial control by enabling stronger bonding, leading to highly organized and complex nanoparticle assemblies with well‐defined geometries. For example, anisotropic core‐satellite clusters have been fabricated by regioselectively incorporating DNA patches at the ends or sides of AuNRs,^[^ ^71^ ^]^ the tips of AuNSs,^[^ ^73^ ^]^ and the edges of AuNTs.^[^ ^72^ ^]^ Regioselectively modified nanoparticles exhibit selective and directional interparticle binding, enabling a wide range of hierarchical assemblies. For instance, monovalent patchy AuNPs can specifically attach to the faces or vertices of multivalent patchy AuNCs, resulting in the formation of 24 distinct complex nanoassemblies (Figure 4e).^[^ ^78^ ^]^ Using DNA barrel‐directed patchy nanoparticles as a unified structure enables the assembly of diverse forms, ranging from anisotropic colloidal clusters to multidimensional crystalline superstructures, including graphene‐like quasi‐2D arrays, which are rarely observed in nanoparticle assemblies.^[^ ^70^ ^]^

## 5 Photonic Nanomaterials Assembled Using DNA Origami

DNA self‐assembly, particularly via DNA origami, offers a robust and precise platform for organizing photonic nanomaterials with unparalleled spatial accuracy. Unlike traditional lithographic methods, DNA‐assembled structures provide superior positional control and nanoscale programmability, enabling the tailored integration of fluorophores and metal nanoparticles. This precise arrangement facilitates tunable optical effects and the emergence of complex photonic phenomena, which are highly beneficial for applications in biological sensing, imaging, and light‐assisted therapy.

### 5.1 Structural DNA Nanotechnology

The successful bottom‐up assembly of photonic nanomaterials requires templates with structural compatibility, precise surface properties, and effective patterning capabilities to integrate photonic components.^[^ ^40^ ^]^ DNA nanostructure templates can provide the stability required to support the construction of photonic building blocks.^[^ ^17^ ^]^ Early attempts to utilize DNA in nanostructure formation resulted in topologically defined architectures. However, these structures exhibited excessive conformational flexibility, limiting their effectiveness in guiding the assembly of well‐defined materials. The development of DNA constructs with sufficient rigidity has been a significant advancement in this field.

**Figure 5:**
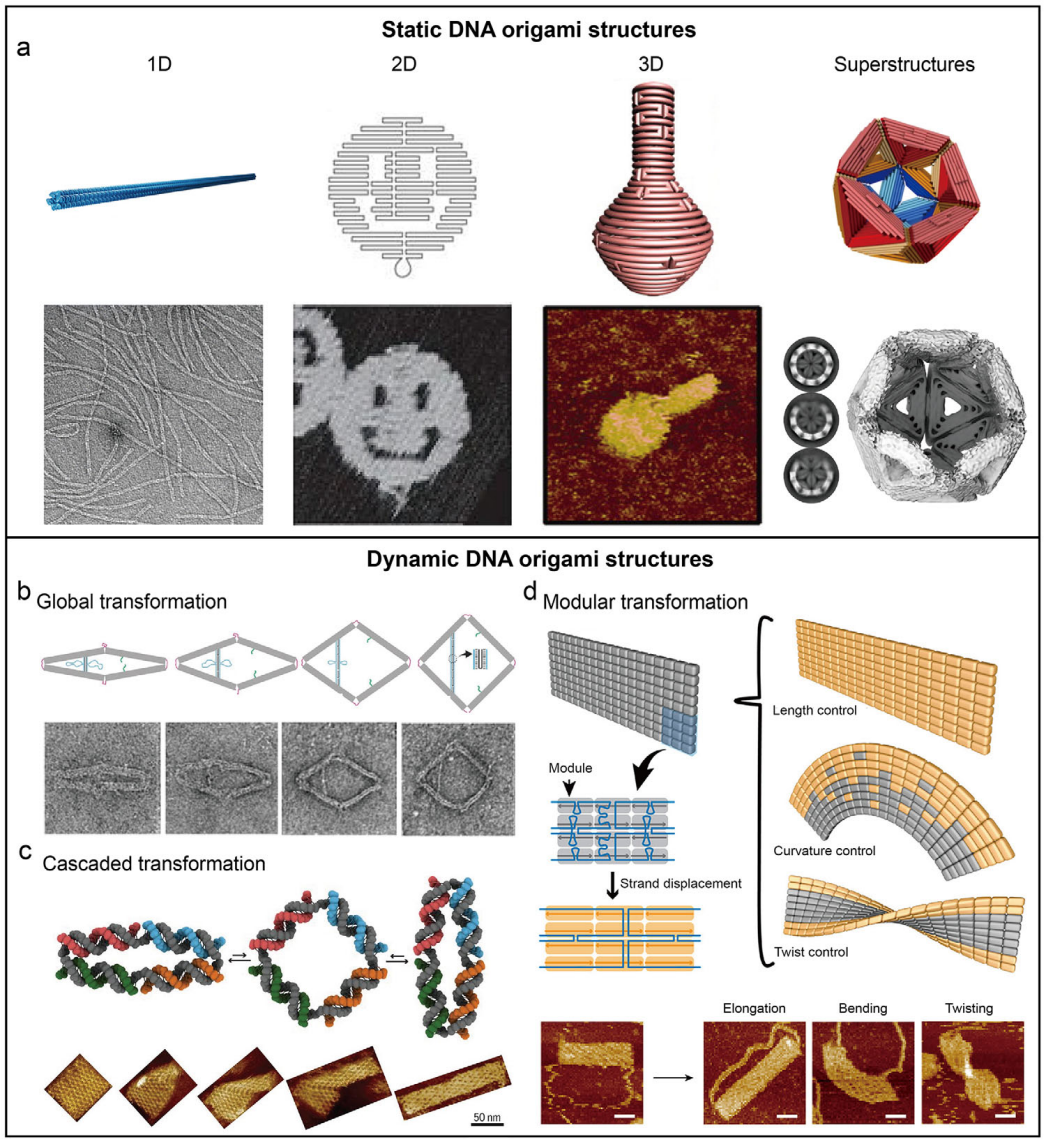
Static and dynamic DNA origami structures. a) Examples of multidimensional static DNA origami structures, including a 1D 6‐helix bundle (6HB)^[^ ^139^ ^]^ (Copyright 2024, Royal Society of Chemistry), a 2D smiley face^[^ ^106^ ^]^ (Copyright 2006, Nature Publishing Group), a 3D nanoflask^[^ ^140^ ^]^ (Copyright 2011, AAAS) and an icosahedral superstructure^[^ ^141^ ^]^ (Copyright 2021, Nature Publishing Group). b) Global transformation of a rhombus‐shaped DNA origami structure.^[^ ^124^ ^]^ Copyright 2016, Nature Publishing Group. c) Cascaded transformation of a DNA domino array.^[^ ^128^ ^]^ Copyright 2017, AAAS d) Modular transformation of a modular expandable origami system. Reproduced with permission.^[^ ^135^ ^]^ Copyright 2021, American Chemical Society.

DNA nanotechnology represents a remarkable frontier in technology, and its development has been extensively reviewed in detail.^[^ ^14^, ^15^, ^101^ ^]^ Briefly, Nadrian Seeman's groundbreaking research in the 1980s established the basis of DNA nanotechnology by introducing immobile junctions, which enabled the construction of double‐crossover molecules.^[^ ^102^ ^]^ This innovation led to the tile strategy,^[^ ^103^ ^]^ facilitating the parallel assembly of defined DNA nanostructures. Subsequent advances expanded the geometric capabilities of DNA assemblies through the development of rigid tiles, while star‐shaped motifs were introduced as versatile building blocks.^[^ ^104^ ^]^ These efforts culminated in the creation of diverse structures, such as DNA tetrahedra and dodecahedra, assembled from motifs with defined vertices and precise angular configurations. A significant leap occurred with the development of single‐stranded tiles,^[^ ^105^ ^]^ which allowed for the construction of large, complex geometries and hollow shapes. The introduction of DNA origami by Rothemund in 2006 brought a significant breakthrough to the field, allowing for the precise construction of nanoscale structures.^[^ ^106^ ^]^ This method employs a long scaffold strand folded by shorter staple strands, yielding a variety of multidimensional arbitrary shapes (**Figure** **5**a).^[^ ^15^ ^]^

In patterning photonic building blocks, DNA origami nanostructures offer unmatched advantages due to their addressability and programmability.^[^ ^15^ ^]^ DNA sequences can be designed to direct the precise arrangement of components within the nanostructure, optimizing interactions and improving device performance. Furthermore, by adjusting the length and configuration of DNA strands, the spacing between photonic building blocks can be fine‐tuned to achieve specific optical properties. This level of control enables the manipulation of photonic device functionality. In addition, large addressable DNA origami structures formed through the polymerization and self‐assembly of DNA origami monomers can accommodate large nanoparticles and form lattices with long‐range order.^[^ ^54^, ^107^, ^108^, ^109^, ^110^, ^111^, ^112^, ^113^, ^114^, ^115^, ^116^ ^]^ However, the removal of oligomers formed by the assembly or polymerization of only a few monomers, along with the unassembled monomers, remains a significant challenge for further applications. It is worth noting that in addition to assembly‐based approaches, DNA‐based photonic materials can be fabricated through direct metallization on DNA origami templates.^[^ ^117^ ^]^ Metal nanoparticles synthesized using DNA origami molds^[^ ^118^ ^]^ or DNA‐assisted lithography^[^ ^119^ ^]^ also hold great potential for photonic applications.

Dynamic, reconfigurable DNA origami nanostructures offer environmental responsiveness,^[^ ^120^ ^]^ enhancing their versatility in fabricating complex photonic nanomaterials with tailored properties.^[^ ^8^, ^22^, ^25^, ^121^ ^]^ The most significant advantage lies in their dynamic controllability of distance and configuration, allowing precise tuning of photonic building block arrangements. This adaptability enables responses to external stimuli, optimizing interactions and enhancing desired optical properties.^[^ ^122^ ^]^ Furthermore, dynamic DNA origami can promote the integration of complex functionalities, thereby broadening the versatility of photonic devices for biomedical applications.^[^ ^34^, ^123^ ^]^ Depending on the connectivity of DNA units, reconfigurable DNA origami structures can be classified into three main types: global transformations,^[^ ^124^, ^125^, ^126^, ^127^ ^]^ cascaded transformations,^[^ ^128^, ^129^, ^130^, ^131^, ^132^, ^133^, ^134^ ^]^ and modular transformations.^[^ ^135^ ^]^ Global transformations occur when all connected DNA units collectively change conformation due to rigid interconnections, allowing coordinated structural adjustments in response to external stimuli (Figure 5b). Cascaded transformations involve sequential conformational changes, where the transformation of one DNA unit triggers the next through switchable connections, akin to a domino effect (Figure 5c). Modular transformations allow independent adjustments of specific units within the structure, providing multiple pathways for shape alteration (Figure 5d). Efforts to combine these different types of transformations have further enhanced the versatility of dynamic DNA origami nanostructures.^[^ ^136^, ^137^, ^138^ ^]^ Despite significant progress, the yield and reversibility of these transformations remain areas that warrant careful attention.

### 5.2 DNA Origami‐Directed Arrangement of Fluorophores

**Figure 6:**
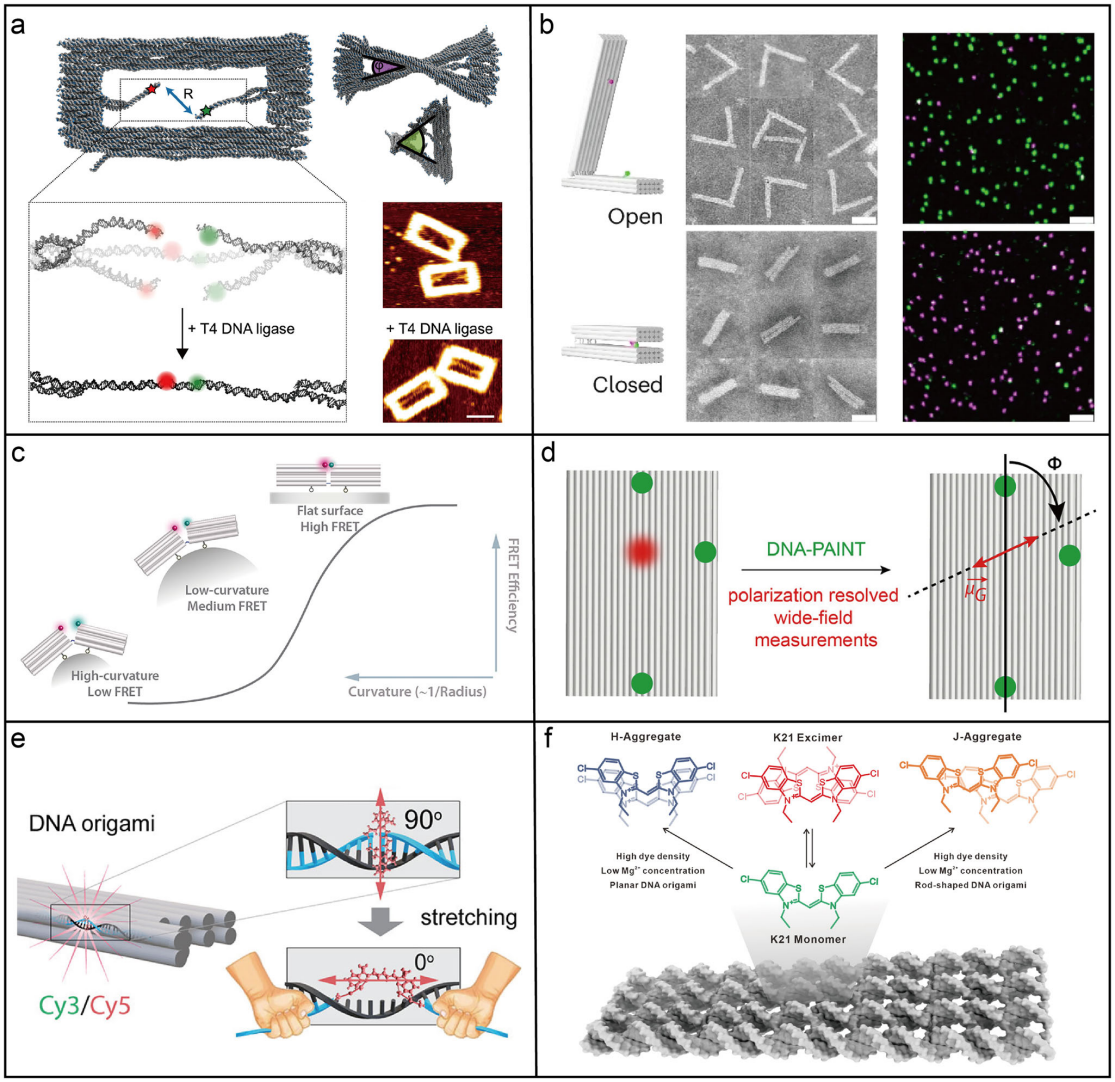
DNA origami‐directed assembly of fluorophores. a) DNA origami platform engineered to replicate a DNA double‐strand break for optical observation of the end‐joining process.^[^ ^145^ ^]^ Copyright 2020, American Chemical Society. b) Dynamic DNA origami nanostructure capable of large conformational changes, engineered to produce a strong optical signal response via FRET.^[^ ^147^ ^]^ Copyright 2024, Nature Publishing Group c) Shape‐adaptable DNA origami system composed of segmented DNA origami units linked by flexible DNA connectors that incorporate a FRET pair.^[^ ^148^ ^]^ Copyright 2023, American Chemical Society. d) 2D rectangular DNA origami structure used to control and study the orientation of single fluorescent molecules.^[^ ^150^ ^]^ Copyright 2021, American Chemical Society. e) Deterministic positioning and orientation of single Cy3 and Cy5 molecules on a DNA origami structure using various incorporation strategies.^[^ ^151^ ^]^ Copyright 2022, American Chemical Society. f) Precise control over the assembly modes of cyanine dye K21 on DNA origami.^[^ ^152^ ^]^ Copyright 2024, American Chemical Society.

DNA origami provides a robust platform for arranging single emitters,^[^ ^142^ ^]^ such as fluorophores, with nanoscale precision in distance,^[^ ^133^, ^143^, ^144^, ^145^, ^146^, ^147^, ^148^ ^]^ orientation,^[^ ^149^, ^150^, ^151^ ^]^ and conformation.^[^ ^152^, ^153^, ^154^ ^]^ This modular and programmed capability allows the development of sophisticated photonic systems, enabling phenomena, such as Förster resonance energy transfer (FRET),^[^ ^155^ ^]^ in which energy is transferred between a donor and acceptor fluorophore pair with high sensitivity to their spatial separation, facilitating distance measurements with sub‐nanometer precision.^[^ ^41^ ^]^ A prominent example of DNA origami‐based FRET distance control involves the study of a DNA double‐strand break and its subsequent end‐joining processes. As depicted in **Figure** **6**a, a DNA origami scaffold was engineered to simulate a DNA double‐strand break, arranging two DNA duplexes labeled with Cy3B (donor) and ATTO647N (acceptor) on opposite sides of the structure.^[^ ^145^ ^]^ Using T4 DNA ligase from bacteriophage to catalyze the ligation of complementary overhangs, the system allowed single‐molecule FRET measurements to monitor the ligation process with sub‐nanometer precision. Beyond FRET, the nanoscale spatial arrangement achieved by DNA origami also influences other photophysical processes, including fluorescence lifetime modulation, plasmonic coupling, and excitonic coherence, broadening its impact on photonic nanomaterials.

Dynamic DNA origami structures often offer large conformational changes that allow for greater and more versatile distance control. This enhances the optical contrast of the output FRET signal. As shown in Figure 6b, a dynamic DNA origami system with two arms fluctuating at an equilibrium angle of ≈90° could transition to a parallel conformation upon introducing complementary ssDNA interactions. ssDNA overhangs facilitated toehold‐mediated opening by complementary strands, while a short toehold overhang permitted reversible reclosing, mimicking receptor‐ligand interactions. The signal transduction component included bright and stable donor (ATTO542) and acceptor (ATTO647N) dyes arranged on opposite arms to form a FRET pair. A substantial conformational shift, along with a transient guiding interaction, was utilized to regulate the orientation of the dyes within the FRET pair. This approach resulted in enhanced FRET contrast, allowing for clear differentiation between single open and closed states.^[^ ^147^ ^]^ Apart from controlling fluorophore distances through global transformations of DNA origami,^[^ ^146^, ^147^ ^]^ cascade transformations can further provide a stepwise allosteric distance change.^[^ ^133^ ^]^

Another innovative application involves curvature‐adaptive DNA origami systems. As depicted in Figure 6c, a shape‐adaptable DNA origami structure was developed, consisting of two 3D blocks connected by flexible DNA linkers arranged in a single plane.^[^ ^148^ ^]^ For signal transduction, a FRET pair consisting of Cy3B as the donor and ATTO647N as the acceptor was strategically placed at the inner edges of the two blocks. When the structure underwent bending due to the curvature of the particle surfaces to which the DNA origami was attached, the separation between the donor and acceptor changed, resulting in fluctuations in FRET efficiency. This working principle enables the system to provide a fundamentally different approach to curvature sensing when combined with single‐molecule FRET readout.

The efficiency of interaction between individual emitters depends not only on their spatial separation but also on their relative orientation.^[^ ^149^ ^]^ Using a 2D rectangular DNA origami structure, fluorophores such as ATTO647N, ATTO643, and Cy5 were spatially positioned and oriented by adjusting binding positions and their immediate surroundings (Figure 6d).^[^ ^150^ ^]^ The three dyes were selected for their key properties: ATTO647N for its brightness and ability to bind to surfaces and DNA, ATTO643 for its reduced nonspecific binding, and Cy5 for its positive charge. In combination with polarization‐resolved excitation measurements and super‐resolution imaging using the nanoscale topography (DNA‐PAINT) technique, the authors revealed that fluorophores, when introduced with additional spacing, tended to bind to the DNA and align in a specific orientation. This alignment was more strongly determined by the surrounding molecular environment than by the nature of the fluorophore itself.

Although the orientation of fluorophores can be determined, orienting individual molecules remains a persistent challenge. As demonstrated in Figure 6e, the relative orientation of individual Cy3 and Cy5 molecules with respect to the DNA origami structure can be systematically controlled by attaching them to two complementary oligonucleotide strands, which are hybridized while leaving certain bases unpaired in the scaffold.^[^ ^151^ ^]^ Specifically, when these fluorophores, attached via dual linkages to ssDNA staples, were hybridized to the origami structure in a defined manner, resulting in varying numbers of unpaired bases, the molecular orientation can be manipulated, spanning from parallel to perpendicular in relation to the dsDNA helix bundle within the origami.

Recently, DNA origami has been used to assemble benzothiazole cyanine dye K21 into well‐defined geometries, replicating the structure of natural light‐harvesting systems through the arrangement of multi‐pigment complexes supported by protein scaffolds.^[^ ^154^ ^]^ DNA templates with various structures, including L‐shaped, zigzag, ring, and T‐junction configurations, acted as stable frameworks for arranging K21 dye molecules into tightly packed aggregates resembling J‐aggregates, which demonstrated strong inter‐monomer coupling. These DNA‐guided dye aggregates functioned as “excitonic wires,” facilitating directional energy transfer over distances of up to half a micrometer. Additionally, the DNA templates were further programmed to introduce geometric complexity, supporting the modular assembly of advanced photonic architectures. It was also observed that K21 exhibited a variety of assembly forms, such as monomers, H‐aggregates, J‐aggregates, and excimers, when integrated with DNA origami (Figure 6f).^[^ ^152^ ^]^ By adjusting factors such as ion concentration, dye concentration, and the configuration of DNA origami, precise control over the assembly modes of K21 was achieved. This tunable aggregation behavior not only governs the electronic coupling between dye molecules but also enables programmable control over emergent photophysical phenomena, such as long‐range exciton transport and tailored light‐matter interactions.^[^ ^41^ ^]^ The integration of these DNA origami‐templated excitonic wires and the DNA‐PAINT technique enabled single‐molecule assessment of hybridization dynamics at docking sites on dye‐loaded rectangular Meta‐DNA,^[^ ^113^ ^]^ assembled 24‐helix bundle (24HB) DNA nanostructures.^[^ ^153^ ^]^

### 5.3 DNA Origami‐Templated Assembly of Metal Nanoparticles

DNA origami provides a programmable platform for the precise arrangement of metal nanoparticles, enabling engineered plasmon coupling, Fano resonances, and plasmonic CD effects in the visible spectrum. For example, the DNA origami 24HB structure, featuring nine helically arranged attachment sites, enabled the precise arrangement of 10‐nm‐diameter AuNPs to study plasmonic coupling.^[^ ^156^ ^]^ Beyond serving as spatial templates, DNA origami‐mediated assembly of metal nanoparticles also allows for the precise modulation of nanoscale photophysical processes, such as plasmonic coupling, chiroptical effects, and exciton‐plasmon interactions. These phenomena arise from DNA origami's unique ability to control nanoparticle placement, orientation, and dynamic rearrangement, thereby facilitating tunable optical responses that are difficult to achieve using conventional fabrication techniques.

For instance, a 14HB DNA origami‐guided system consisting of two AuNPs separated by an interspersed silver nanoparticle (AgNP) exhibited plasmonic coupling between the AuNPs, facilitated by the AgNP with minimal energy loss. This system further illustrated full control over the spatial organization of heterogeneous nanoparticles using DNA origami templates.^[^ ^157^ ^]^ The distance‐dependent CD transfer efficiency has also been systematically explored using AuNPs and AuNRs with varying aspect ratios, demonstrating the versatility of DNA origami as a spatial template.^[^ ^39^ ^]^ Beyond nanometer‐scale distances, DNA origami enables chiral transfer over larger scales. A DNA origami platform, with an overall length of 100 nm, positioned two AuNRs (54 nm long, 23 nm wide) at its ends with a 90° tilt and a 62‐nm surface‐to‐surface distance. A 40 nm AuNP was placed between the rods, creating an L‐shaped configuration that exhibited chiral plasmonic effects over extended distances.^[^ ^158^ ^]^

**Figure 7:**
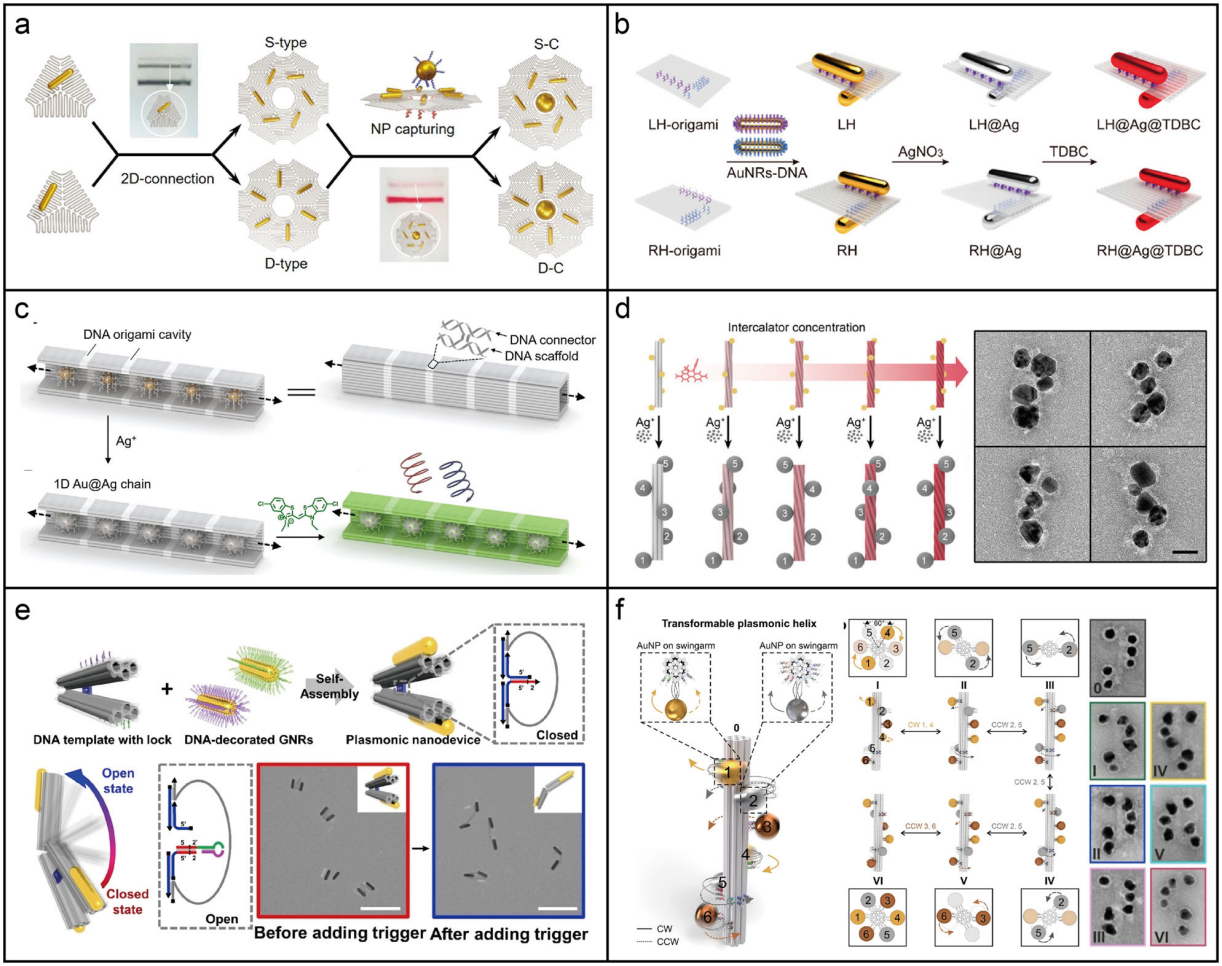
DNA origami‐templated assembly of metal nanoparticles. a) Chiral satellite‐core superstructure with a central AuNP at its core, encircled by six AuNRs arranged in a spiral configuration on a 2D hexagonal DNA origami sheet, followed by gel purification of the resulting spirals and superstructures.^[^ ^160^ ^]^ Copyright 2022, American Chemical Society. b) Chiral plasmonic‐excitonic hybrids consisting of an AuNR dimer with asymmetric arrangement, coated with a silver layer, and incorporated into an excitonic matrix.^[^ ^162^ ^]^ Copyright 2021, American Chemical Society. c) Synthesis of 1D Au@Ag chains via DNA‐directed assembly, followed by in situ Ag shell deposition and hierarchical chiral interaction with cyanine dye K21.^[^ ^108^ ^]^ Copyright 2022, Wiley. d) Chemo‐mechanical control of chiroptical responses using DNA origami deformed by DNA intercalators, with signal amplification through Ag enhancement.^[^ ^163^ ^]^ Copyright 2024, American Chemical Society. e) Plasmonic nanodevice with an asymmetric configuration of AuNRs assembled on a tweezer‐like DNA origami template, where DNA locks hold the device in a closed state, and DNA key structures trigger its opening via strand displacement reactions. Reproduced with permission.^[^ ^172^ ^]^ Copyright 2022, Wiley. f) Transformable plasmonic helix with six AuNPs on DNA origami swingarms, capable of transitioning between six states from left‐ to right‐handed helicity via cooperative rearrangement of the AuNPs. Reproduced with permission.^[^ ^186^ ^]^ Copyright 2023, Wiley.

In addition to individual DNA origami structures, DNA origami can self‐assemble into larger frameworks for directing nanoparticle assembly. Employing a self‐assembled 2D supramolecular template based on truncated‐triangular DNA origami with a side length of 80 nm and a thickness of 2.5 nm, six distinct types of 2D AuNR superstructures were constructed, classified into bi‐stars (3 types) and pinwheels (3 types). These superstructures, with a uniform size (≈240 nm) and precisely controlled by the DNA template, featured double‐ or mono‐layered geometries and exhibited significant anisotropy.^[^ ^159^ ^]^ Building upon this, a chiral satellite‐core nanoparticle superstructure was created using a 2D hexagonal DNA origami sheet, where a central AuNP was surrounded by six AuNRs.^[^ ^160^ ^]^ Specifically, extended ssDNA strands on triangular DNA origami captured and positioned AuNRs functionalized with complementary strands. This process led to the formation of S‐type or D‐type spirals within the hexagonal 2D arrangement of the origami sheets. Additionally, a distinct DNA‐functionalized AuNP was positioned at the center of the AuNR spiral, creating conformation‐modulated chiral plasmonic superstructures with satellite‐core configurations (**Figure** **7**a). Recently, a combined approach of DNA origami self‐assembly and electron beam lithography (EBL) enabled the precise positioning of AuNPs on a SiO₂ surface for plasmonic metasurfaces.^[^ ^161^ ^]^ In this approach, DNA origami structures were initially immobilized onto the EBL‐patterned substrate, directing the selective attachment of AuNPs through DNA hybridization. A subsequent sol‐gel process encapsulated the DNA within a silica shell, thereby improving its structural integrity. This method yielded high percentages of individual nanospheres (74%) and dimers/trimers (65% and 60%, respectively), with a spatial precision of 9 nm. The resulting metasurfaces demonstrated optical responses that could be adjusted based on the polarization of light.

DNA origami has also been employed in chiral plasmonic‐excitonic hybrids to investigate light‐matter interactions. For instance, planar DNA origami templates have been employed to assemble asymmetric AuNR dimers coated with a silver layer and an excitonic matrix.^[^ ^162^ ^]^ As shown in Figure 7b, two AuNRs (≈40 × 10 nm) were positioned on the opposing surfaces of a planar DNA origami sheet (90 × 60 × 2 nm), creating a 90° twist and generating 3D conformational chirality. Following the in situ reduction of Ag shells, the excitonic compound 5,6‐Dichloro‐2‐[[5,6‐dichloro‐1‐ethyl‐3‐(4‐sulfobutyl)‐benzimidazol‐2‐ylidene]‐propenyl]‐1‐ethyl‐3‐(4‐sulfobutyl)‐benzimidazolium hydroxide inner salt (TDBC), which is known for forming J‐aggregates in aqueous solution, was incorporated via electrostatic interactions due to its exceptional optical properties. Hierarchical chiral hybrid superstructures of chromophores and nanoparticles exhibit unique chiroptical responses due to complex chiral interactions. As illustrated in Figure 7c, DNA‐capped AuNPs were positioned within a DNA origami cavity using complementary capture strands. Sequence‐specific DNA connectors between the cavities then facilitated the self‐assembly of the AuNPs into a 1D chain superstructure. This assembled structure was subsequently coated with an Ag shell and functionalized with K21 dyes, which aggregate into J‐aggregates when inserted into the minor grooves of the DNA. The resulting hybrid structure exhibited hierarchical chirality across multiple scales, ranging from sub‐nanometer to micrometer dimensions.^[^ ^108^ ^]^

Besides controlling the placement of nanoparticles using the addressability of DNA staples, the chemo‐mechanical changes in DNA origami triggered by DNA intercalators has been proposed as an innovative method for modulating chiroptical responses.^[^ ^163^ ^]^ Intercalators like ethidium bromide induce unwinding of dsDNA, causing torsional strain within the DNA origami structure, which subsequently affects the nanoparticle arrangement. As depicted in Figure 7d, the chiroptical response can be finely tuned by adjusting the concentration of intercalators. To enhance this effect, Ag enhancement was employed to amplify the chiroptical signals by enlarging the nanoparticle size, while concurrently stabilizing the structure by reinforcing the 6HB DNA origami template. Furthermore, the sensitivity of the chiroptical signals to changes in intercalator concentration could be influenced by several factors, including the specific intercalator used, the combination of different intercalators, and the structural rigidity of the DNA origami structures. This approach uniquely leverages the ability of DNA origami to control both the spatial arrangement of nanoparticles and the intercalator‐induced torsional deformation of its framework, enabling precise manipulation of CD signals at the nanoscale.

A key advantage of structural DNA nanotechnology is its ability to dynamically control the spatiotemporal conformations of photonic nanomaterials in response to environmental signals. Two primary approaches are typically employed to control the dynamics of DNA origami‐templated photonic architectures. The first approach involves switchable transformations of DNA origami templates in response to external stimuli, such as DNA,^[^ ^164^, ^165^, ^166^, ^167^, ^168^, ^169^, ^170^, ^171^, ^172^, ^173^ ^]^ RNA,^[^ ^174^ ^]^ aptamers,^[^ ^175^, ^176^, ^177^ ^]^ temperature,^[^ ^176^ ^]^ pH,^[^ ^178^, ^179^ ^]^ light^[^ ^180^ ^]^ and others,^[^ ^172^, ^181^ ^]^ primarily operating under equilibrium conditions. The second approach focuses on driving the movement of individual nanoparticles to align with or dynamically rearrange on DNA origami templates.^[^ ^182^, ^183^, ^184^, ^185^, ^186^ ^]^ A notable example of switchable DNA origami transformation is shown in Figure 7e, where a responsive DNA origami‐based plasmonic nanodevice amplifies trace signals into detectable CD responses.^[^ ^172^ ^]^ In this approach, two AuNRs were incorporated into a dynamic DNA origami scaffold, creating a 3D, tweezer‐like chiral plasmonic nanostructure that produced intense CD signals when exposed to circularly polarized light. DNA locks, created from folding strands, connected the tweezer arms and kept the device in a closed state. Upon introducing molecular inputs and utilizing a DNA circuit for signal amplification, the nanodevice opened, triggering an amplified CD signal that mimicked biological signaling cascades. Figure 7f illustrates a transformable plasmonic helix with swinging AuNPs. In this configuration, multiple AuNPs were systematically repositioned along a shared DNA origami shaft.^[^ ^186^ ^]^ The 24HB DNA origami shaft provided 16 binding sites in six rows, spaced 8.4 nm apart, for AuNP attachment via DNA footholds. DNA swingarms, extending from the origami, guided AuNPs between binding sites, enabling large‐leap translocations. Through sequence‐specific DNA interactions, the DNA‐coated AuNPs were reversibly translocated in either a clockwise or counterclockwise direction, allowing programmable transitions between different states.

### 5.4 DNA Origami‐Guided Co‐Assembly of Optical Emitters and Metal Nanoparticles

Plasmonic nanostructures can enhance spontaneous emission, modulate emission polarization, and provide precise control over the radiation pattern of quantum emitters. A key challenge in experimental implementation, however, lies in the accurate positioning of a single emitter within the nanoscale hotspot of a plasmonic antenna. DNA origami provides a powerful solution to this challenge by precisely controlling the local optical environment at the nanoscale, enabling the manipulation of light‐matter interactions fundamental to many photophysical processes. This precise spatial arrangement capability of DNA origami allows for the accurate placement of optical emitters, such as fluorophores^[^ ^36^ ^]^ and Raman chromophores,^[^ ^37^ ^]^ between metal nanoparticles, significantly enhancing fluorescence^[^ ^28^, ^35^, ^36^, ^187^ ^]^ and SERS signals.^[^ ^37^, ^38^ ^]^ This makes it highly effective for single‐molecule sensing applications.^[^ ^188^, ^189^ ^]^

#### 5.4.1 Photonic Nanomaterials for Enhanced Fluorescence Spectroscopy

A 3D pillar‐shaped DNA origami structure (220 nm length, 15 nm core diameter, and 30 nm base diameter) was employed to attach two AuNPs with docking sites for a single ATTO647N dye molecule in the gap.^[^ ^190^ ^]^ This configuration resulted in fluorescence enhancement up to 117‐fold for the dye molecule placed within the 23‐nm gap between two 100‐nm AuNPs. Using a similar pillar‐shaped DNA origami template, hybrid constructs were fabricated by incorporating the natural light‐harvesting complex, peridinin‐chlorophyll α‐protein, and coupling it with dimer optical antennas. This design resulted in a fluorescence enhancement of up to 500‐fold.^[^ ^191^ ^]^ The influence of critical parameters, including the position of individual dyes,^[^ ^192^ ^]^ nanoparticle stoichiometry,^[^ ^193^ ^]^ interparticle distance,^[^ ^194^ ^]^ and the material composition of AuNPs^[^ ^195^, ^196^ ^]^ on the emission spectrum of dyes was experimentally investigated. Additionally, optical antennas composed of two 60‐nm AuNPs, separated by a two‐layer rectangular DNA origami sheet (≈50 × 60 × 5 nm) and with a single Cy5 fluorophore positioned near the center of the gap, were shown to make both the excitation and emission of individual, rotatable fluorophores highly directional.^[^ ^197^ ^]^ The adaptability of structural DNA nanotechnology has been further highlighted in a dynamic light‐matter interaction framework, where a single Atto647N molecule autonomously navigated in a unidirectional manner toward the hotspot of a plasmonic dimer antenna, illustrating the potential of DNA nanotechnology for the creation of controllable and dynamic photonic systems.^[^ ^198^ ^]^

**Figure 8:**
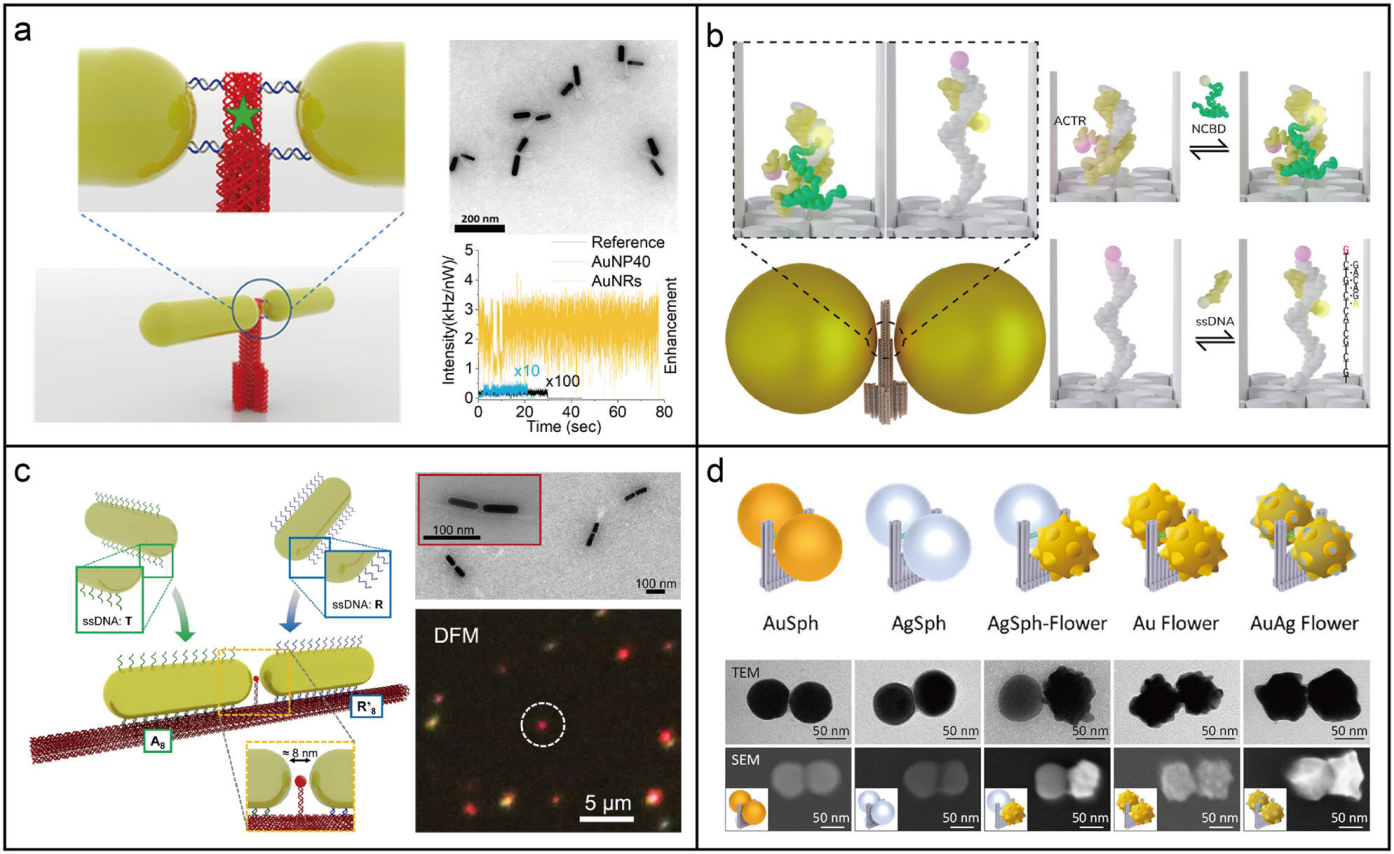
DNA origami‐guided co‐assembly of optical emitters and metal nanoparticles. a) The configuration of AuNRs into a plasmonic dimer, resulting in fluorescence enhancement of a NIR dye by as much as 1600 times at the plasmonic hotspot.^[^ ^200^ ^]^ Copyright 2023, American Chemical Society. b) DNA origami nanoantennas with 100 nm AuNPs enhance photon count rates and enable the observation of fast biomolecular dynamics.^[^ ^201^ ^]^ Copyright 2024, American Chemical Society. c) DNA origami‐assisted tip‐to‐tip alignment of AuNR dimers with accessible interparticle gaps, featuring a plasmonic hotspot for SERS analyte detection, validated by dark‐field microscopy.^[^ ^220^ ^]^ Copyright 2023, Nature Publishing Group. d) Versatile assembly of plasmonic DNA origami nanoantennas using a DNA origami nanofork design with AuNPs or AgNPs to assess SERS performance.^[^ ^224^ ^]^ Copyright 2023, American Chemical Society.

A photoluminescence enhancement of up to 42 times was observed in a DNA‐assisted dimer nanoantenna. This system consisted of a triangular DNA origami framework and two DNA‐functionalized AuNPs, maintaining a nominal surface‐to‐surface gap of ≈3 nm.^[^ ^199^ ^]^ As shown in **Figure** **8**a, a plasmonic nanoantenna dimer based on AuNRs was assembled using a 3D DNA origami framework, which provided a ≈6 × 12 nm gap between the two pillars. This configuration facilitated fluorescence amplification in the near‐infrared (NIR) spectrum for dyes such as Cy7, ATTO 740, and Alexa Fluor 750, leading to an enhancement of up to 1600 times.^[^ ^200^ ^]^ In addition to monitoring single fluorophores, DNA origami nanoantennas have demonstrated significantly improved photon detection rates and single‐molecule FRET dynamics. Recently, DNA nanoantennas incorporating FRET pairs spanning the red and NIR spectral regions were used to enhance photon count rates and monitor rapid biomolecular interactions within the narrow gap between two plasmonic nanoparticles (Figure 8b).^[^ ^201^ ^]^ Specifically, the interaction between the nuclear coactivator binding domain of the CBP/p300 transcription factor (NCBD) and the activation domain of SRC‐3 (ACTR) was examined using a FRET pair of AlexaFluor 647 and LD750. This system demonstrated the creation of transient encounter complexes, which persisted for durations approximately on the scale of 100 µs. Additionally, the hybridization of short ssDNA to its complementary sequence was investigated using the FRET pair of Dy‐751 and Cy5B. This research revealed a transition path time of 17 µs at photon count rates ≈10 MHz, representing a significant improvement by one order of magnitude compared to the existing state of the art. These advancements demonstrate the potential of DNA origami‐templated nanoantennas for studying ultrafast biomolecular dynamics with unprecedented precision.

Furthermore, significant advancements have enabled the precise lateral positioning of single molecules within sub‐5 nm gaps between plasmonic structures, exhibiting with strong optical confinement. This was accomplished by constructing a nanocavity, consisting of an AuNP positioned on top of a gold film, separated by a nanoscale spacer made from a two‐layer DNA origami plate. By positioning a single Cy5 molecule at the center of this nanocavity, fluorescence enhancements exceeding 4000‐fold with a high quantum yield (≥50%) were achieved.^[^ ^202^ ^]^ Further exploration of the interaction between ultrafast laser pulses and a single ATTO647 molecule, strongly coupled to such a sub‐wavelength plasmonic nanocavity at room temperature, revealed the possibility of observing cavity quantum phenomena in molecular systems at ambient conditions.^[^ ^203^ ^]^

#### 5.4.2 Photonic Nanomaterials for Surface‐Enhanced Raman Spectroscopy

SERS is a technique that is highly sensitive to surface interactions, leveraging metal nanostructures to greatly amplify the Raman scattering signals of molecules that are adsorbed, thus allowing for ultra‐sensitive molecular detection and analysis. Despite its potential, SERS often faces reproducibility challenges due to the inherently small Raman scattering cross‐sections of molecules compared to fluorescence. Signal enhancement occurs in plasmonic hotspots, where intense electromagnetic fields are localized. In recent decades, significant attention has been devoted to synthesizing plasmonic nanostructures with reduced gaps to achieve more reliable SERS signals.^[^ ^204^, ^205^ ^]^ DNA origami offers a precise platform for creating plasmonic structures and accurately positioning single molecules within the hotspots, addressing the challenges of random molecular adsorption.^[^ ^37^ ^]^ The geometry of the plasmonic nanostructures and the dimensions of the gaps between particles significantly affect the SERS enhancement factor.

DNA origami triangles were initially proposed as substrates for SERS by arranging two AuNPs into dimers, creating intense Raman scattering hotspots within the interparticle gaps. By optimizing both the nanoparticle size and the interparticle gap size, strong field enhancements were achieved. The accurate placement of a specific number of Raman reporter molecules facilitated the detection of unique Raman signals from individual TAMRA molecules bound to the DNA origami substrates.^[^ ^206^ ^]^ The assembled plasmonic system, comprising 40 nm AuNP dimers with an average gap of 3.3 ± 1 nm on a 40 × 45 nm^2^ DNA origami platform, achieved local field enhancements by several orders of magnitude. This underscores the effectiveness of DNA origami as a robust and dependable method for efficiently generating SERS‐active nanoparticle assemblies with gap sizes smaller than 5 nm.^[^ ^207^ ^]^ Moreover, the programmable assembly capabilities of DNA origami allow for the integration of larger nanoparticles and the construction of assemblies containing greater numbers of nanoparticles, further expanding the versatility of SERS‐active systems.^[^ ^112^, ^208^ ^]^ For example, using the rhombus‐shaped super‐origami nanostructure as a template, a nanocluster composed of four spatially arranged 80‐nm AuNPs exhibited distinct peak‐and‐dip Fano characteristics. The significant enhancement at the Fano minimum wavelength enabled the detection of a strong SERS spectrum from even a single dye molecule.^[^ ^112^ ^]^

Compared to spherical AuNP dimers,^[^ ^209^, ^210^, ^211^, ^212^, ^213^, ^214^ ^]^ DNA origami‐templated AuNS,^[^ ^215^, ^216^ ^]^ Au@Ag nanostars^[^ ^216^, ^217^, ^218^ ^]^ and AuNT^[^ ^219^ ^]^ dimers exhibit stronger localized plasmonic fields at their sharp‐edged tips. The enhanced fields result in superior electric field enhancement, leading to excellent SERS efficiency. For AuNRs with elongated cylindrical shapes, the orientation of DNA origami‐templated dimers plays a crucial role in a crucial role in SERS signal amplification.^[^ ^220^, ^221^, ^222^ ^]^ As shown in Figure 8c, the 14HB DNA origami facilitated the accurate alignment of AuNR dimers with their tips positioned at an average distance of 8 nm, large enough to accommodate smaller proteins from the solution.^[^ ^220^ ^]^ According to finite difference time domain (FDTD) simulations, the maximum field enhancement for nanorod dimers exceeded that of nanospheres at the center of the nanoantenna gap for all interparticle distances between 2 and 10 nm. This enhancement is attributed to the superior longitudinal plasmonic mode of AuNRs, resulting from their increased tip curvature‐to‐volume ratio and reduced surface plasmon damping, compared to spherical AuNPs. Moreover, the unique features of AuNCs, such as their vertices, edges, and faces, allow for the formation of a wider range of nanostructure configurations with varying nanogaps, in contrast to spherical AuNPs. These configurations can significantly amplify electromagnetic fields within the nanogaps.^[^ ^67^ ^]^ Using a DNA origami triangular template, a pattern recognition strategy was employed to assemble AuNCs into plasmonic nanostructures with high reproducibility.^[^ ^223^ ^]^ By adjusting the positioning and quantity of capture strands on the DNA origami scaffold, various geometric structures with nanometer‐level precision and controllable gaps were fabricated. These structures included face‐to‐face dimers, face‐to‐side dimers, side‐to‐side dimers, face‐to‐face trimers, and face‐to‐face tetramers. The meticulously designed AuNC nanostructures enabled the study of Raman enhancement within localized hotspot regions, serving as an ideal model for single‐molecule SERS. When a single Raman probe molecule was positioned within these nanogaps, the significant electromagnetic field enhancements in the hotspots resulted in amplified single‐molecule SERS signals.

Beyond nanoparticle shapes, the influence of nanoparticle composition on the SERS performance of plasmonic DNA origami nanoantennas has been systematically investigated. This study utilized DNA origami nanoforks combined with spherical and anisotropic Au or Ag nanoparticles to examine their impact on SERS enhancement.^[^ ^224^ ^]^ As shown in Figure 8d, five different plasmonic nanoparticle dimer designs were used, followed by extensive characterizations of the SERS performance on both the fully dye‐coated dimers and dimers with single dye molecules. The results clearly demonstrated that the use of anisotropic particles was particularly advantageous, as they induced multiple hotspots at the edges, surpassing the SERS intensity achieved by spherical dimers. By integrating a Ag nanoparticle with a Au particle within a single dimer configuration, broadband excitation was achieved, effectively spanning nearly the full visible spectrum. Of all these configurations, the most adaptable plasmonic dimer for SERS applications paired a spherical AgNP with an Au nanoflower, delivering significant enhancement over a wide range of wavelengths.

While DNA origami‐templated assembly offers remarkable precision in nanomaterial design, several challenges require attention. In the case of DNA origami‐directed arrangement of fluorophores, issues related to fluorophore efficiency and spatial control may arise. Ensuring precise spatial alignment on the scaffold is challenging, as even minor misalignments can affect energy transfer or emission spectra. For DNA origami‐templated assembly of metal nanoparticles, achieving nanoparticle stability and effective DNA functionalization can be complicated. Photonic metal nanoparticles are sensitive to environmental conditions,^[^ ^7^ ^]^ making stable DNA attachment difficult. For DNA origami‐guided co‐assembly of optical emitters and metal nanoparticles, the proximity of the components may lead to undesirable interactions, such as non‐radiative energy transfer, reducing the overall optical efficiency. Beyond nanoscale precision, scalability and reproducibility in larger‐scale production remain significant challenges, as even slight variations can affect the consistency of the final product. Additionally, the long‐term stability of the entire structure is determined by the stability of the DNA origami itself, which is often influenced by temperature and ionic concentration.

## 6 DNA‐Assembled Photonic Nanomaterials for Diagnostic Applications

The analysis of biological processes and dynamic biochemical reactions demands critical attributes, including high sensitivity, low limits of detection (LOD), high specificity for accurate discrimination in complex biofluids, and rapid test‐to‐answer times suitable for routine diagnostic use. DNA‐assembled nanoparticles, with their unique and tunable optical properties, such as localized surface plasmon resonance (LSPR),^[^ ^98^, ^99^, ^100^ ^]^ optical activity,^[^ ^122^ ^]^ enhanced fluorescence^[^ ^36^ ^]^ and SERS,^[^ ^37^ ^]^ are ideal candidates for developing advanced optical nanoprobes with broad applications in biological sensing.^[^ ^26^ ^]^ The programmability of DNA allows for the precise conversion of molecular recognition or binding events into optical signals via a signal transducer, enabling rapid analysis and improving both accuracy and specificity. This capability makes DNA‐assembled photonic nanomaterials powerful tools for next‐generation diagnostics, bridging the gap between molecular biology and advanced photonic technologies.

### 6.1 Biological Sensing

#### 6.1.1 Chirality‐Based Biosensing

While biological molecules typically exhibit CD responses in the UV range, plasmonic nanoparticles arranged in chiral geometries can produce CD responses that are spectrally engineerable across a broad range.^[^ ^33^ ^]^ The plasmonic chirality not only enhances sensitivity, but also simplifies detection, and enables real‐time biosensing, making these nanostructures a powerful tool for advanced sensing technologies.^[^ ^26^ ^]^

The bioanalytical potential of DNA‐assembled chiral superstructures was demonstrated by arranging AuNRs in side‐by‐side patterns with a defined twist angle between their axes.^[^ ^225^ ^]^ This configuration resulted in pronounced polarization rotation and chiral responses, aligning with theoretical predictions. The DNA‐assembled chiral system exhibited excellent signal linearity with target DNA concentrations, achieving a LOD as low as 3.7 aM. This chiroplasmonic approach is especially beneficial for the detection of large biological molecules. Furthermore, twisted side‐by‐side AuNR dimers bound by ssDNA were constructed for the selective and specific recognition of miRNA sequences in living cells.^[^ ^226^ ^]^ The dynamic assembly of AuNRs within living cells was validated through signal changes in chiroptical signals, enabling the real‐time measurement of biomarkers in living cells.

**Figure 9:**
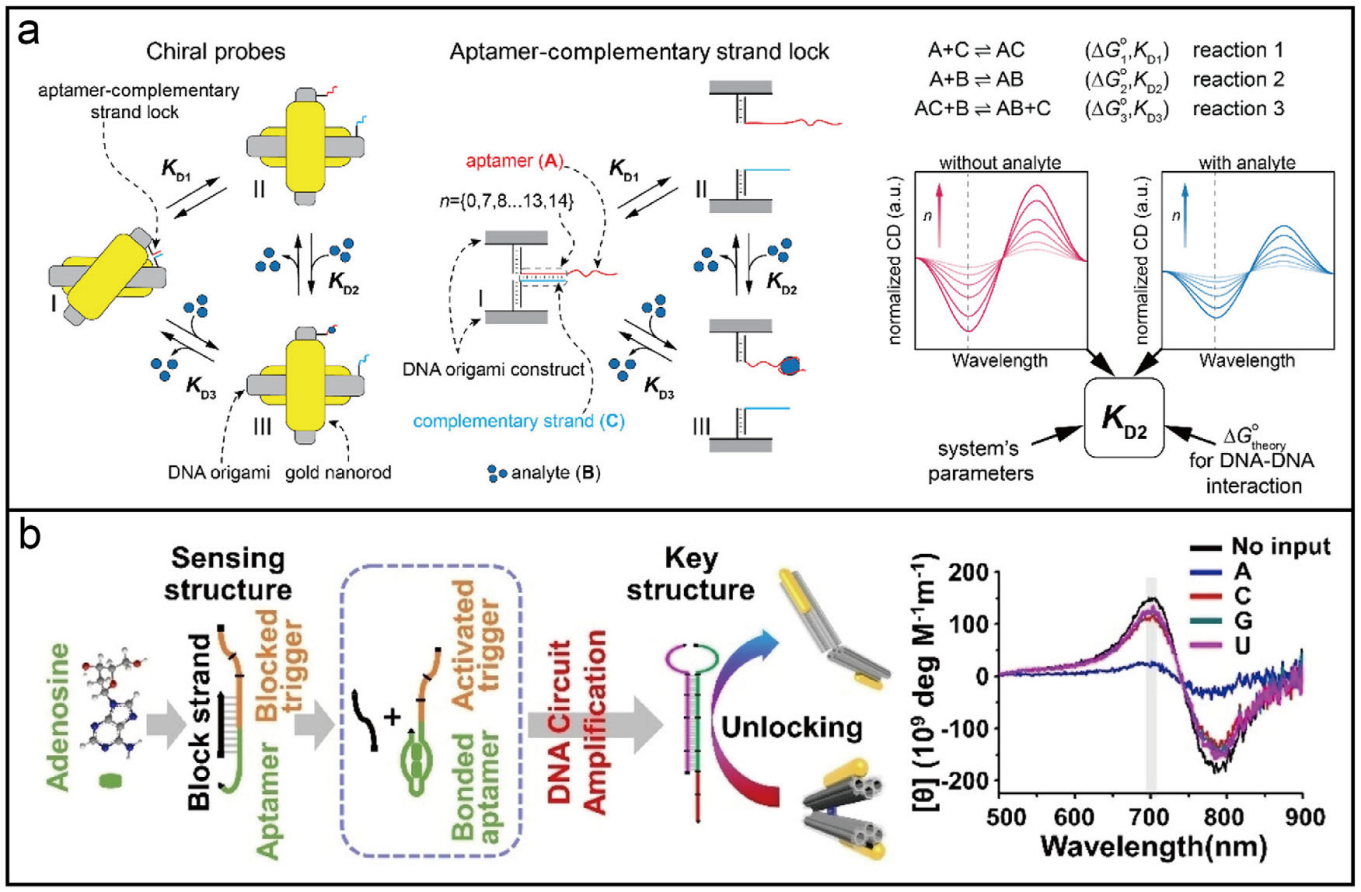
Chirality‐based biosensing. a) Schematic of a DNA origami‐assembled chiral plasmonic probe with an aptamer and complementary strand as an analyte‐responsive lock, where hybridization length controls the transition between open and closed configurations.^[^ ^177^ ^]^ Copyright 2022, American Chemical Society. b) Schematic illustration and CD signal changes of the adenosine‐triggered cascade DNA amplification circuit for unlocking plasmonic nanodevices.^[^ ^172^ ^]^ Copyright 2022, Wiley.

In addition to twisted stacks of nanorods assembled by DNA, cross‐shaped DNA nanostructures created using DNA origami templates have been utilized for detecting viral RNA sequences. Reconfigurable DNA origami templates were employed to assemble AuNRs into cross dimers, generating strong CD signals. Structural transformations were triggered by the addition of specific nucleic acid sequences, enabling chirality to switch in different states. Consequently, target RNA sequences, such as those from the hepatitis C virus genome, were successfully detected at concentrations as low as 100 pM by monitoring changes in CD signals.^[^ ^174^ ^]^ This reconfigurable DNA origami‐based chiral plasmonic sensing device was further extended to detect various analytes. For instance, a DNA aptamer was integrated into the system to detect adenosine.^[^ ^175^ ^]^ In the absence of adenosine, the DNA origami structure remained locked by the aptamer, and the AuNR dimer retained its chiral configuration. Upon the addition of adenosine, the aptamer recognized the analyte and opened the origami, resulting in a structural transformation to the relaxed state. This transition triggered a distinct plasmonic CD response, providing a straightforward method for optical detection. Building on this strategy, DNA origami‐based plasmonic chiral assemblies, combined with a thermodynamic model based on competitive hybridization reactions, were developed to characterize the affinity and specificity of aptamer‐analyte pairs (**Figure** **9**a).^[^ ^177^ ^]^ By varying the hybridization lengths of DNA, the chemical equilibrium of the chiral probes shifted toward the hybridized, locked configuration. Changes in the amplitude of CD spectra were used to calculate the equilibrium dissociation constant (K_D_​), enabling the characterization of low, intermediate, and high K_D_ ranges. In addition to sequence‐specific responses, DNA aptamers binding to distinct thrombin recognition sites were employed to construct a plasmonic chiroptical sensor.^[^ ^181^ ^]^ Thrombin‐binding aptamers TBA and HD22 were positioned at opposite ends of each origami bundle, with AuNRs assembled at the top and bottom of the origami arms in an initial “open” configuration. Upon the introduction of thrombin, the aptamers recognized different epitopes of the protein, transitioning the plasmonic metamolecule into an X‐shaped chiral state, resulting in a detectable CD signal change. This strategy achieved thrombin detection with a K_D_ as low as 1.4 nm, owing to the cooperative interaction between the two aptamers.

Despite the success of reconfigurable DNA origami‐based chiral plasmonic sensors as proof‐of‐concept devices, their low detection sensitivity for biological signal molecules remained a significant challenge in physiological environments. To address this limitation, signal amplification strategies have been integrated to enhance the interactions between messenger molecules and nanodevices. For instance, catalytic hairpin assembly, an isothermal nucleic acid amplification method, was incorporated into DNA origami‐based plasmonic sensors, achieving a detection limit as low as 10 pm for tumor marker RNA sequences, an improvement of one order of magnitude compared to previous approaches.^[^ ^227^ ^]^ This integration of internal signal amplification circuits, coupled with plasmonic CD output, demonstrates the potential for advanced biological detection applications. As shown in Figure 9b, two AuNRs were assembled on a tweezer‐shaped DNA origami structure containing a DNA lock that fixed the plasmonic nanodevice in a closed state, producing strong CD signals.^[^ ^172^ ^]^ Cascade DNA circuits were integrated into the system, serving as recognition and amplification elements for a variety of inputs such as nucleic acids, adenosine, chiral tyrosinamides, or specific tumor cell receptors. Upon exposure to low concentrations of target molecules, the plasmonic nanodevices underwent conformational changes, transitioning from a closed to an open state and producing distinct CD signal outputs. These smart nanodevices offer promising capabilities for in situ bioanalyses, particularly with further advancements to enhance their responsiveness to pathophysiological biomarkers. Such improvements could pave the way for their application in precision diagnostics and real‐time monitoring of biological processes.

#### 6.1.2 Fluorescence‐Based Biosensing

Fluorescence‐based detection is a cornerstone of biological analyses due to the wide commercial availability of fluorophores, accurate quantification capabilities, high sensitivity, and ease of operation. However, a significant challenge in DNA‐based fluorescent biosensors is that natural nucleic acids exhibit little to no fluorescence. This limitation is addressed by attaching fluorophores at defined positions on DNA strands through specific interactions. Furthermore, when nanoparticles are used as quenchers, the intensity of the fluorescent signal becomes dependent on the distance between the nanoparticles and fluorophores, enabling distance‐dependent signal modulation.^[^ ^228^ ^]^

One innovative example is a DNAzyme motor developed for intracellular operation that responded to specific molecular targets within living cells. In this system, 20 nm AuNPs were functionalized with hundreds of substrate strands and multiple DNAzyme molecules, which were initially inhibited by locked strands. Upon recognition of target molecules, strand displacement reactions unlocked the DNAzyme, which then hybridized with the substrate strands. This reaction released fluorescently labeled DNA strands, producing fluorescent signals. The motor was self‐powered, with each step leading to an increase in fluorescence, enabling diverse applications in controlling and regulating biological functions.^[^ ^229^ ^]^ To improve the biostability of DNA motors, a DNA motor system incorporating a degradable MnO_2_ nanosheet was designed. The substrate and enzyme strands of a Mn^2+^‐dependent RNA‐cleaving DNAzyme were conjugated to AuNPs and assembled with MnO_2_ nanosheets. Upon entering the cell, glutathione (GSH) reduced MnO_2_, releasing AuNPs and generating localized Mn^2+^ concentrations. In the presence of the target molecule, strand displacement activated the enzyme strands, leading to substrate cleavage and the released fluorescein, producing fluorescent signals. This system utilized GSH consumption to trigger a hybridization‐cleavage‐release cycle, protecting the DNA motor from GSH‐induced degradation and enhancing its biostability. Fluorescence changes allowed real‐time monitoring of the DNA motor's activity. This design was successfully used for amplified imaging and detection of *survivin* mRNA in live cells and mice with tumors. It is worth noting that the motion of micronized DNA motors in response to targeted analytes can be recorded using a smartphone, eliminating the need for a dedicated microscope.^[^ ^230^, ^231^ ^]^ This development paves the way for low‐cost, portable diagnostic tools with potential applications in point‐of‐care settings.

**Figure 10:**
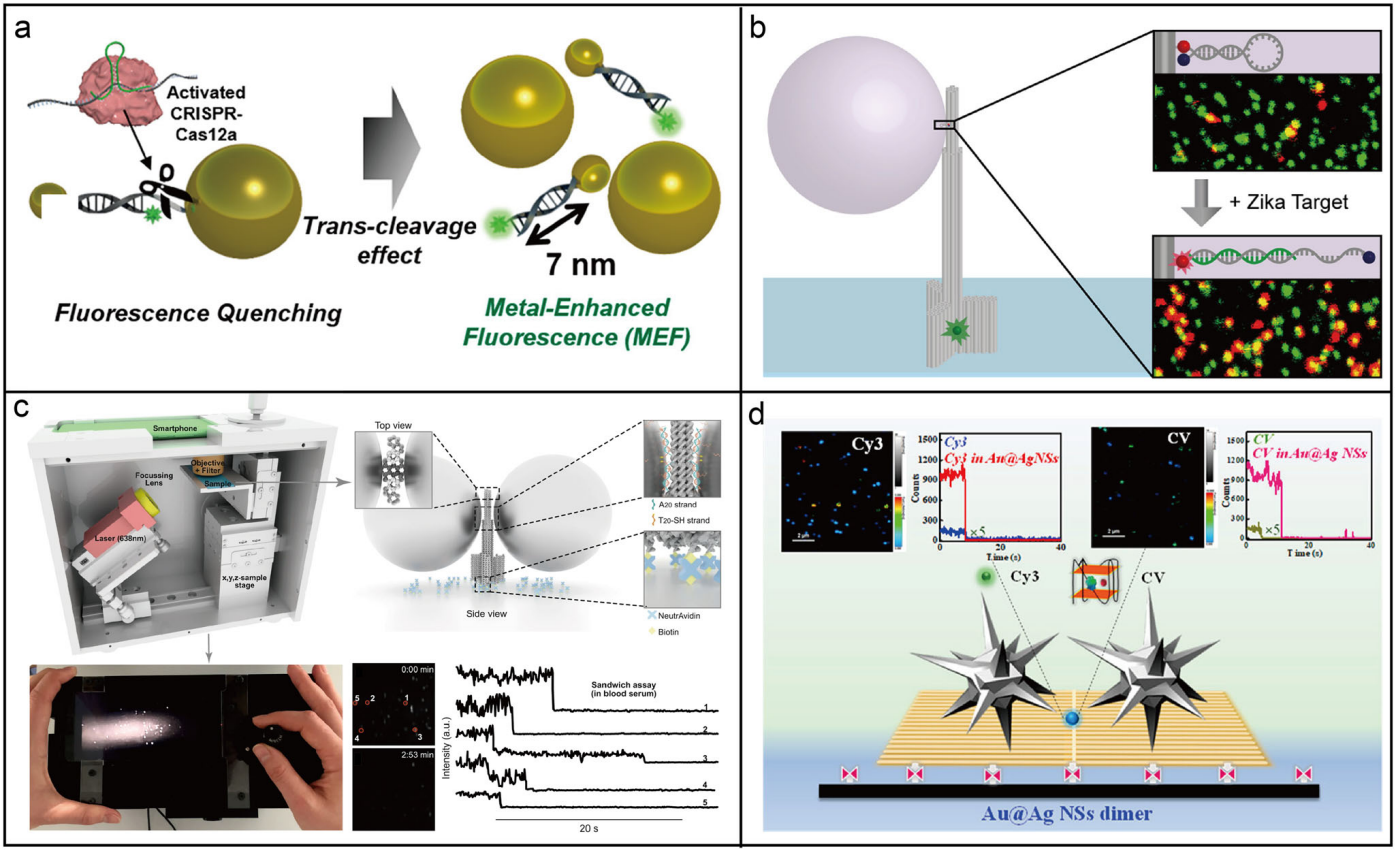
Fluorescence‐based biosensing. a) Cell‐free DNA detection assay using DNA‐functionalized AuNPs with CRISPR‐Cas12a.^[^ ^232^ ^]^ Copyright 2021, American Chemical Society. b) Fluorescence enhancement‐based detection of specific Zika virus DNA and RNA sequences using DNA origami scaffolds to position a fluorescence‐quenching hairpin near a plasmonic nanoparticle.^[^ ^234^ ^]^ Copyright 2017, American Chemical Society. c) Addressable NanoAntennas with cleared hotspots scaffolded by DNA origami, enhancing single‐emitter brightness up to 461‐fold and enabling single‐molecule detection on a portable smartphone microscope.^[^ ^235^ ^]^ Copyright 2021, Nature Publishing Group. d) DNA origami‐arranged bimetallic Au@Ag nanostars, enabling up to 65‐fold fluorescence enhancement of Cy3 dye and 42‐fold enhancement of crystal violet dye.^[^ ^196^ ^]^ Copyright 2023, American Chemical Society.

To reduce false‐positive outputs and enhance the sensitivity, CRISPR‐Cas12a was integrated into a detection platform for cell‐free DNA (cfDNA) without the need for nucleic acid amplification (**Figure** **10**a).^[^ ^232^ ^]^ Two differently sized AuNPs were connected by a 9 nm long ssDNA strand and a complementary 7 nm long ssDNA strand. One end of the latter was immobilized on the smaller AuNP, while the other end, labeled with fluorescein isothiocyanate (FITC), remained free and in proximity to the larger AuNPs, resulting in a quenching state. In the presence of target cfDNA, the non‐hybridized ssDNA was cleaved by the trans‐cleavage activity of the activated CRISPR‐Cas12a complex, causing the dissociation of AuNPs and fluorescence recovery. This fluorescence intensity was proportional to the target cfDNA concentration, enabling highly sensitive detection without nucleic acid amplification. Using this system, BRCA‐1, a breast cancer gene, could be detected within 30 min, achieving femtomolar sensitivity. This rapid, selective sensor holds great promise for detecting a wide range of nucleic acid biomarkers.

The precise addressability of DNA origami enables the arrangement of fluorophores to study dipolar interactions between multiple fluorophores and to allow fine‐tuning of their optical properties. For example, DNA structures featuring a central opening, tagged with a pair of FRET dyes, were temporarily attached to a nanocapillary tip, enabling optical responses to voltage variations.^[^ ^233^ ^]^ When a higher ionic current was applied, the system progressively induced distortions and regulated structural alterations, which were tracked by variations in FRET efficiency. By repositioning a single dye, the voltage sensitivity of the sensor was fine‐tuned, showcasing its adaptability and multifunctionality. This sensing device holds potential for imaging transmembrane potentials in live cells. To further improve the signal‐to‐noise ratios and simplify detection, a DNA origami beacon was constructed, consisting of two rectangular panels connected by a foot at the bottom.^[^ ^146^ ^]^ One panel was designed to arrange donor fluorophores, while the other positioned acceptor fluorophores, forming a multi‐FRET pair fluorophore network. This configuration provided a high‐output signal, enabling the detection of small DNA sequences at concentrations as low as 100 pm within 45 min using a standard fluorescence microscope. The compact and efficient design of this biosensor broadens its application for portable microscopy and point‐of‐care diagnostic tools.

Fluorescence can also be enhanced by placing emitters in the near‐fields of metal nanostructures. Tinnefeld and colleagues constructed DNA origami‐assembled optical antennas that exploited plasmonic hotspots created by metal nanoparticles to enhance fluorescence signals.^[^ ^36^ ^]^ They systematically investigated the influence of factors, including the size, number, shape, and material, as well as the distance and orientation between the dye and nanoparticle, applying these insights to create effective sensing platforms. Single 80 nm AgNP nanoantennas were constructed, as shown in Figure 10b, employing a molecular beacon‐like structure to amplify the signal of a single fluorescent dye by several orders of magnitude. The fluorescence signal was selectively enhanced only in the presence of specific target nucleic acids, enabling highly sensitive detection. Using this platform, artificial DNA and RNA sequences specific to the Zika virus were detected in both buffer solutions and heat‐inactivated human serum.^[^ ^234^ ^]^ To simplify point‐of‐care diagnostics, a cost‐effective trident NanoAntennas with cleared hotspots (NACHOS) design was developed, featuring two pillars for attaching AgNPs (Figure 10c).^[^ ^235^ ^]^ At the bifurcation between the two pillars, three capture strands were positioned within the plasmonic hotspot. Upon hybridization with DNA target strands specific to Klebsiella pneumonia, the hotspot region was exposed to an imager strand, resulting in fluorescence enhancements of up to 461‐fold (average of 89 ± 7‐fold). This setup enabled detection using a standard smartphone camera paired with an inexpensive ($8 USD) objective lens, demonstrating the potential for mobile, point‐of‐care applications. To validate its broader applicability, a portable, battery‐powered smartphone microscope was developed and successfully used to perform single‐molecule detection of DNA specific to antibiotic‐resistant Klebsiella pneumonia. Very recently, method targeting a 151‐nucleotide sequence unique to antibiotic‐resistant *Klebsiella pneumoniae* was developed, enabling single‐molecule fluorescence detection of non‐amplified DNA at attomolar concentrations using the trident NACHOS system.^[^ ^236^ ^]^ The assay employed a compact, cost‐efficient microscope with a large field of view and integrated microfluidic flow to enhance target capture efficiency. Fluorescence enhancement was achieved through dense arrays of DNA origami NanoAntennas fabricated using nanosphere lithography and site‐specific DNA origami placement.^[^ ^237^ ^]^ The system exhibited the capability to identify 200 ± 50 molecules from a sample of 600 in just 100 µL within an hour, achieving a level of sensitivity similar to that of polymerase chain reaction, but without the need for molecular amplification. Furthermore, similar sensitivity was demonstrated in untreated human blood plasma, broadening its compatibility with complex biological samples and enabling the detection of shorter nucleic acid fragments not amenable to traditional amplification methods. This DNA origami‐based platform represents a scalable, cost‐effective solution for highly sensitive nucleic acid detection, with broad applicability in diverse diagnostic and healthcare settings.

DNA‐assembled plasmonic nanoantennas not only quantify biomolecules but also enable detailed investigations of biomolecular interactions by significantly increasing photon count rates. As a proof of concept, this approach was used to analyze two intrinsically disordered proteins and the hybridization of a short ssDNA strand with its complementary strand within plasmonic hotspots.^[^ ^201^ ^]^ This approach yielded single‐molecule FRET time traces with an order‐of‐magnitude improvement compared to conventional methods, revealing coupled folding and binding dynamics. The enhanced sensitivity to fast biomolecular dynamics bridges the time scales of molecular dynamics simulations, enabling the investigation of previously unobservable ultrafast biophysical processes.

Beyond spherical nanoparticles, bimetallic Au@Ag nanostars were arranged on rectangular DNA origami with a single Cy3 dye positioned at the interparticle gap, achieving fluorescence enhancement of up to 65‐fold (Figure 10d).^[^ ^196^ ^]^ The presence of ATP induced the aptamer to form a G‐quadruplex structure, further amplifying the fluorescence signal by ≈42‐fold. This dual enhancement mechanism enabled ATP detection at the single‐molecule level, demonstrating the potential of DNA‐assembled plasmonic nanoantennas for high‐sensitivity biosensing applications.

#### 6.1.3 SERS‐Based Biosensing

DNA assembly enhances the design and tunability of plasmonic nanostructures, enabling the creation of substrates with strong local electromagnetic fields that significantly expand SERS applications in biosensing. MicroRNAs, such as miRNA‐10b, serve as critical biomarkers for early pancreatic cancer detection, helping to distinguish pancreatic ductal adenocarcinoma (PDAC) from chronic pancreatitis (CP) and normal controls (NC). A duplex‐specific nuclease (DSN)‐assisted dual‐SERS biosensor was developed to detect miRNA‐10b in exosome and plasma samples using Fe_3_O_4_@Ag‐DNA‐Au@Ag@DTNB (SERS tag) conjugates. Target miRNA hybridized with complementary DNA probes, and the DSN enzyme cleaved the DNA probe in the DNA/miRNA duplex, releasing SERS tags and quenching the SERS signal. The released miRNA was recycled, amplifying the detection signal. This one‐step assay achieves a LOD as low as 1 aM with single‐base resolution. When applied to plasma samples from PDAC, CP, and NC, the biosensor demonstrated significant SERS signal differences, underscoring its potential for point‐of‐care cancer diagnostics.^[^ ^238^ ^]^ The same group subsequently developed locked DNA‐modified Au@DTNB for in situ detection of exosomal miRNAs directly from serum samples. Upon entering exosomes, the target miRNA bridged Au@DTNB particles, forming plasmonic hotspots that induced strong SERS signals. To enrich exosomes, Fe_3_O_4_@TiO_2_ nanoparticles were incorporated into the sensor, eliminating the need for capture antibodies or ultracentrifugation pretreatment. This target‐triggered hotspot SERS platform detected miRNA‐10b in situ with a LOD of 0.21 fM, offering a robust solution for non‐invasive diagnostics. Additionally, SERS‐based biosensing has been expanded through a DNA structure‐stabilized liquid‐liquid self‐assembled ordered AuNP interface.^[^ ^87^, ^239^ ^]^ When combined with an exonuclease III‐assisted DNA recycling amplification strategy, this liquid‐phase SERS biosensor achieved efficient detection of miRNA‐155 with a LOD of 1.45 fM.^[^ ^87^ ^]^

Rapid and precise identification of various cross‐species disease‐related markers from a single biological sample is essential for efficient disease diagnosis and monitoring. One strategy to achieve this involves employing an array of SERS sensors, each functionalized with distinct capture probes. For example, a DNA tetrahedron‐based SERS sensor utilizing an immobilized AgNR array was developed to detect multiple miRNAs and carcinoembryonic antigen in human serum. Six ssDNA strands self‐assembled into a multiple‐armed tetrahedral DNA nanostructure, capable of hybridizing with three capture DNA strands. This design enabled detection of single, dual, and triple biomarkers in human serum.^[^ ^240^ ^]^ Another approach involves immobilizing a mixture of probe molecules on a single SERS sensor. Asymmetric core‐shell Au@Au@Ag nanoparticles were synthesized and modified with sequences complementary to miRNA‐21, miRNA‐126, and miRNA‐1246. Three distinct Raman probes were adsorbed onto the nanoparticles, while biotinylated DNA capture sequences were immobilized on streptavidin‐modified magnetic beads. In the presence of target miRNAs, a sandwich configuration was formed, where miRNAs hybridized with both the nanoparticles and capture probes, achieving femtomolar detection limits with three characteristic SERS peaks. The detection limits were 1.076 fM for miRNA‐21, 0.068 fM for miRNA‐126, and 4.57 fM for miRNA‐1246. This SERS sensor was further validated to detect miRNA‐21 in 20% human serum and exosome extraction solutions, demonstrating its applicability in complex biological environments.^[^ ^241^ ^]^

**Figure 11:**
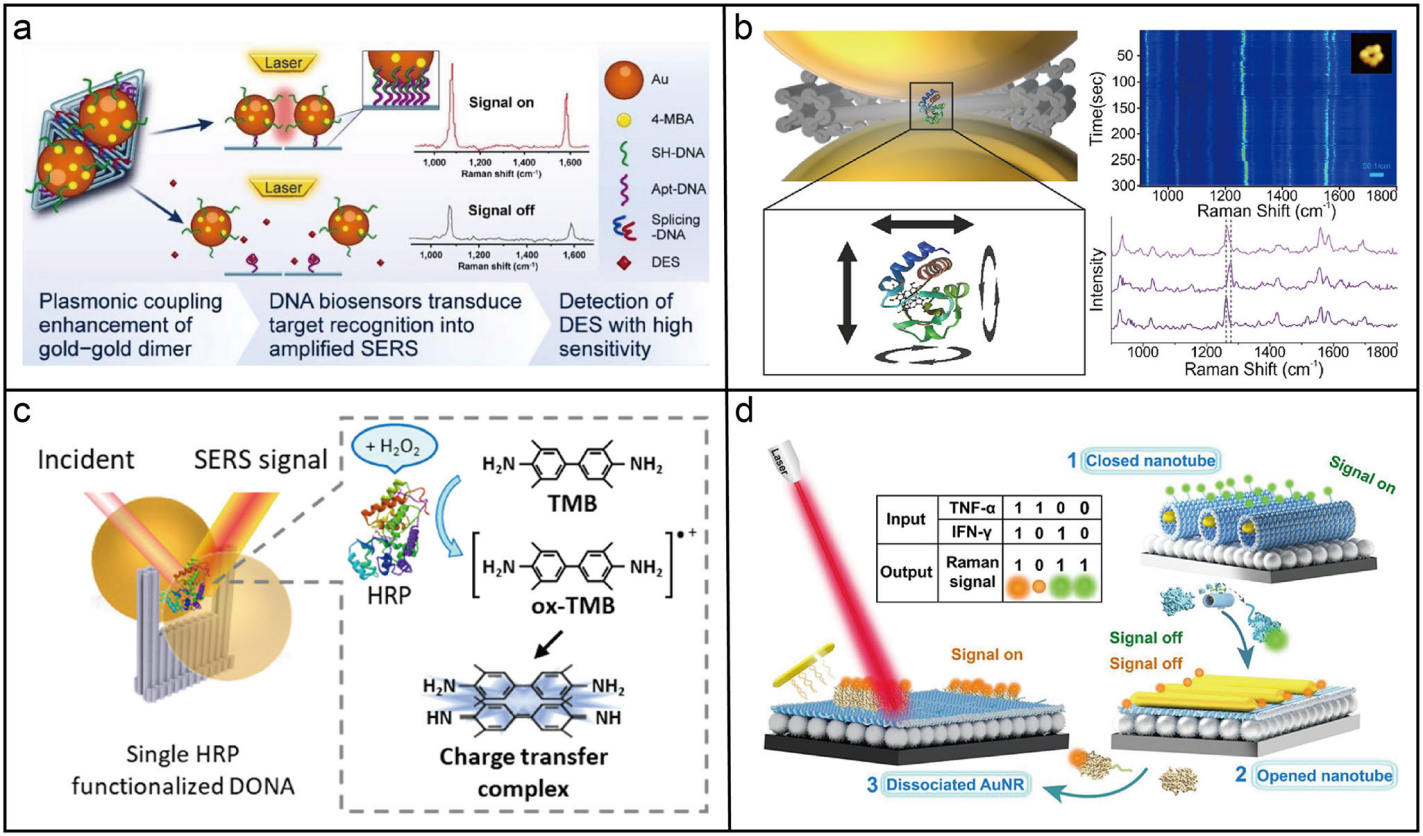
SERS‐based biosensing. a) Plasmonic dimer nanoantennas assembled through DNA origami for use as SERS biosensors in the sensitive detection of diethylstilbestrol at trace levels.^[^ ^208^ ^]^ Copyright 2023, Elsevier. b) Single‐molecule SERS using DNA origami nanoantennas provides insights into cytochrome C structure, conformational dynamics, and oxidation processes.^[^ ^211^ ^]^ Copyright 2024, American Chemical Society. c) Detection of single‐enzyme catalytic reactions using DNA origami‐based plasmonic antennas.^[^ ^212^ ^]^ Copyright 2024, American Chemical Society. d) DNA origami plasmonic nanoantenna for programmable SERS detection of cytokine release syndrome‐associated cytokines through logic‐gated nanotube opening.^[^ ^242^ ^]^ Copyright 2024, American Chemical Society.

Like fluorescence‐based biosensors, precise control over nanogaps, material types, and analyte localization in hotspots plays a crucial role in influencing SERS signals. By leveraging the capabilities of DNA origami, which allows for the precise arrangement of nanoparticle patterns with nanometer accuracy, AuNPs decorated with Raman molecules were assembled on aptamer‐functionalized rhombic DNA origami to construct plasmonic dimers (**Figure** **11**a). These biosensors, due to their high target‐binding affinity, transduced the recognition of diethylstilbestrol into dynamic structural changes in plasmonic nanoantennas, which then generated enhanced Raman signals. A wide linear detection range spanning from 10⁻¹⁰ to 10⁻⁵ M was achieved, with a LOD of 0.217 nm.^[^ ^208^ ^]^ Compared to isotropic nanoparticles, sharp‐tipped nanoparticles enhance SERS due to the tip effect. For instance, bimetallic Au@Ag nanostar dimers with controlled nanogaps, coupled plasmonically, were constructed utilizing rectangular DNA origami. These nanoantennas combined the plasmonic properties of Ag with the chemical stability of Au, serving as efficient SERS substrates for the ultrasensitive detection of pyocyanin, achieving a detection limit of 335 pm, well below the clinical range.^[^ ^217^ ^]^ Furthermore, DNA origami‐directed trimer assemblies demonstrated exceptional sensitivity in detecting dopamine, achieving a detection limit in the picomolar range.^[^ ^216^ ^]^ Additionally, the DNA origami platform facilitated the assembly of Au@Ag nanostar dimers with adjustable nanogaps, allowing for the accurate placement of individual fluorescein, cyanine, and Texas Red dye molecules at the core of two nanostars. These hybrid nanoantennas significantly amplified single‐molecule Raman signals for all three dye molecules, with the enhancement occurring predominantly at the plasmonic hotspot. The enhancement factors, spanning from 10⁹ to 10¹⁰, were observed under both resonant and nonresonant excitation conditions, leading to enhanced photostability during time‐series measurements. The potential of these nanoantennas was demonstrated by successfully detecting a single thrombin protein molecule, selectively placed in a 10 nm‐wide nanogap. From single‐molecule SERS spectra, distinct Raman vibrational signature peaks of the single thrombin protein were recorded.^[^ ^218^ ^]^ To address challenges associated with detecting proteins that lack chromophores and exceed the conventional 2–3 nm size limit for single‐molecule SERS measurements, DNA origami‐assembled AuNR dimers with tunable nanogaps ranging from 3 to 10 nm were recently developed. These dimer assemblies enabled the label‐free detection of single biomolecules, including the Epidermal Growth Factor Receptor (EGFR), a clinically significant cancer biomarker. By allowing specific placement of EGFR within the plasmonic hotspot, this approach achieved single‐molecule sensitivity using label‐free SERS detection, showcasing its potential for clinical diagnostics.^[^ ^222^ ^]^

Apart from mere protein detection, DNA origami‐based SERS sensors also provide valuable insights into protein dynamics, conformational changes, and single‐enzyme catalytic reactions. As a proof of concept, cytochrome C was covalently attached to a DNA staple strand positioned within the adjustable gap of DNA origami‐assembled nanoantennas, optimizing the SERS hotspot distance.^[^ ^211^ ^]^ Shifts in the Amide III band were used to characterize oxidation processes, identifying reduced and oxidized states, and elucidating protein structural changes and chemical modifications at the single‐molecule level (Figure 11b). To enable real‐time monitoring of enzymatic kinetics under catalytic conditions, a single horseradish peroxidase (HRP) molecule was positioned on the nanofork of a plasmonic antenna.^[^ ^212^ ^]^ As shown in Figure 11c, the introduction of hydrogen peroxide (H_2_O_2_) induced Raman peak shifts indicative of HRP oxidation. In the presence of the substrate TMB, time‐series single‐molecule SERS spectra of HRP were recorded, demonstrating its catalytic capacity of a single enzyme. The DNA origami‐directed SERS biosensors are thus powerful tools for probing enzyme activity and advancing the development of plasmon‐enhanced biocatalysis systems.

The DNA origami‐directed SERS biosensors also hold great potential for multiplexed detection. Recently, a stimuli‐responsive DNA origami‐assembled plasmonic nanoantenna was designed to monitor multiple cytokines relevant to cancer immunotherapy (Figure 11d).^[^ ^242^ ^]^ This nanoantenna utilized plasmonic nanotubes, formed by attaching AuNR‐loaded DNA origami nanosheets along their edges and affixing them to an AgNP‐modified silicon wafer. Cytokines, including tumor necrosis factor‐α (TNF‐α) and interferon‐γ (IFN‐γ), induced the opening of the nanotubes and caused the AuNRs to detach from the origami structure upon binding with their respective aptamers. This mechanism facilitated the construction of a full set of Boolean logic gates, which interpreted the cytokine molecules as inputs, with changes in Raman signals serving as the corresponding outputs. The system allowed for the quantification of TNF‐α and IFN‐γ in the serum of tumor‐bearing mice undergoing various immunotherapy treatments.

A dual‐signal method combining CD and SERS has been developed for the quantification of biomolecules in living cells, offering ultrasensitive, accurate, and reliable diagnostic capabilities for clinical diseases. A self‐assembly strategy using Y‐DNA hybridization was employed to construct chiral AuNP core‐satellite structures with high regional controllability. By varying the DNA quantity on AuNPs, a dual‐layer satellite architecture was constructed using a Y‐DNA scaffold. This core‐satellite arrangement featured 30 nm AuNPs as the central core, while 5 and 10 nm AuNPs formed the first and second satellite layers, respectively. These structures exhibited structural chirality and encoded SERS tags. This design enabled strong, tunable CD and SERS signals for monitoring miRNA in living cells. Target miRNA binding caused significant decreases in both CD and Raman signals, achieving detection limits of 0.0051 amol ngRNA⁻¹ for CD and 2.81 × 10⁻^2^ amol ngRNA⁻¹ for Raman, respectively.^[^ ^90^ ^]^

### 6.2 Biological Imaging

High spatiotemporal resolution bioimaging of active molecules and metabolites in vivo is vital for unraveling molecular mechanisms in living cells and biological heterogeneity. Such insights pave the way for advanced diagnostic and therapeutic strategies. DNA‐directed photonic nanomaterials offer exceptional signal enhancement and precise targeting, facilitating real‐time visualization of biomolecular interactions and disease markers for improved diagnosis and therapeutic monitoring. For example, label‐free DNA‐assembled AuNP‐AuNR structures demonstrate sufficient field enhancement for imaging small metabolites in living HeLa cells.^[^ ^71^ ^]^ This supports real‐time probing of organelle environments, emphasizing the potential of structural optimization for multitarget metabolic monitoring and transformative optics applications.

**Figure 12:**
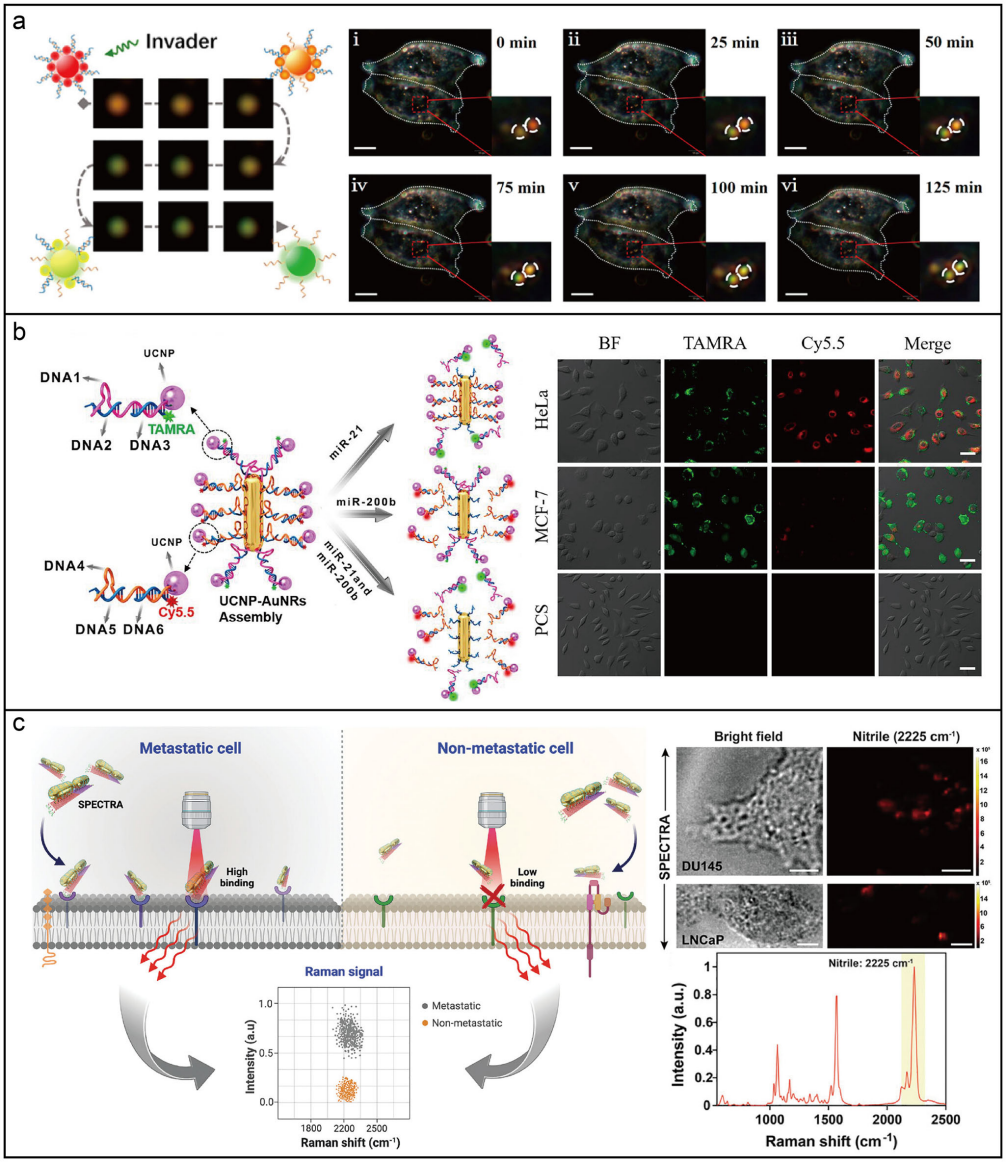
Biological imaging using DNA‐directed photonic nanomaterials. a) DNA‐directed core‐satellite plasmon rulers combined with DFM for real‐time, single‐molecule bioimaging of toehold‐mediated strand displacement kinetics in vitro and in vivo.^[^ ^243^ ^]^ Copyright 2018, American Chemical Society. b) DNA‐bridged AuNR‐UCNP assemblies for multiplexed detection and fluorescence imaging of miRNA‐21 and miRNA‐200b with zeptomolar sensitivity.^[^ ^89^ ^]^ Copyright 2019, National Academy of Sciences. c) DNA origami‐based nanoprobe for targeted Raman imaging of metastatic cells.^[^ ^221^ ^]^ Copyright 2024, Wiley.

LSPR exhibited by DNA‐assembled photonic nanomaterials provide enhanced spatial resolution and enable precise in vivo single‐particle bioimaging. As shown in **Figure** **12**a, DNA‐assembled core‐satellite AuNP structures were synthesized and characterized using dark‐field microscopy to investigate dynamic biophysical processes in living cells.^[^ ^243^ ^]^ The assemblies underwent toehold‐mediated strand displacement, resulting in a detectable blue shift in the scattering wavelength, which could be monitored in real‐time. Using miRNA‐21 as the invading strand, strand displacement kinetics were analyzed at the single‐molecule level, revealing key differences between DNA/RNA and DNA/DNA duplexes. This method enabled the study of molecular kinetics in vivo at low concentrations and allowed accurate detection of miRNA‐21 expression in living cells, advancing the field of single‐structure bioimaging. A multifunctional plasmonic core‐satellite nanoprobe was subsequently developed for integrated cancer diagnosis and therapy.^[^ ^244^ ^]^ This nanoprobe, loaded with doxorubicin (DOX), combines miRNA‐21 detection, drug release, and therapy monitoring within a single platform, enabling miRNA detection, drug‐induced apoptosis, and real‐time therapy evaluation.

Furthermore, DNA‐assembled AuNRs and upconverting nanoparticles (UCNPs) were developed for the multiplexed detection and imaging of miRNA cancer markers, miRNA‐21, and miRNA‐200b (Figure 12b).^[^ ^89^ ^]^ The core‐satellite structure facilitated energy transfer between UCNPs and fluorescent dyes via LSPRs, with energy upconversion efficiently exciting the dyes, achieving zeptomolar sensitivity. Upon miRNA hybridization, the DNA bridge was disrupted, disassembling the nanostructure and altering LSPR signals, enabling precise miRNA quantification. Detection limits reached 3.2 zmol/ngRNA for miRNA‐21 and 10.3 zmol/ngRNA for miRNA‐200b, respectively. This system enables real‐time multiplexed imaging of miRNAs in living cells and in vivo, offering a robust platform for advanced diagnostics, quantitative epigenetics, and personalized medicine. Additionally, two arrays of donor and acceptor fluorophores formed a multifluorophore FRET pair to generate high‐output signals. DNA origami arranged the fluorophores into arrays, enhancing the signal‐to‐noise ratio and enabling target DNA detection at concentrations as low as 100 pM within 45 min using conventional fluorescence microscopy.^[^ ^146^ ^]^ Beyond biomolecule imaging, the dynamic assembly of chiral nanorod structures was validated via FRET fluorescence in living cells.^[^ ^226^ ^]^

DNA‐directed photonic nanomaterials functionalized with targeting ligands have garnered great interest for SERS‐based bioimaging. Unlike individual fluorescence‐based bioimaging, a dual‐signal platform combining SERS and UCNPs enables ultrasensitive, quantitative detection of miRNA‐21 and telomerase in living cells. Using a molecular beacon, miRNA‐21 detection resulted in decreased Raman signals, while telomerase activation enhanced luminescence, allowing precise biomolecular imaging with LODs of 0.011 amol/ngRNA and 3.2 × 10⁻¹^3^ IU.^[^ ^245^ ^]^ To improve the kinetics and sensitivity of nonenzymatic isothermal amplification for microRNA detection, a DNA tetrahedron‐mediated branched catalytic hairpin assembly strategy was developed.^[^ ^246^ ^]^ By tethering hairpin probes to a DNA tetrahedron, reaction kinetics was accelerated 11.1‐fold. When combined with SERS hotspots and Raman reporter‐functionalized AuNPs, this system enabled ultrasensitive in situ imaging of miRNA‐21 in living cells. Additionally, molecular logic gates facilitated multiplexed biomarker analysis, advancing both bioanalysis and clinical diagnosis. SERS probes have also been functionalized with aptamers for in situ tracking of target biological events.^[^ ^53^ ^]^ Using such a nanoprobe, not only intracellular molecules could be imaged,^[^ ^247^ ^]^ but also dual intercellular signal transductions, such as membrane protein dimerization.^[^ ^248^ ^]^ Moreover, a novel core‐satellite gold nanostructure combining dual ratiometric SERS and photoacoustic (PA) imaging enabled precise real‐time detection of H₂O₂, a key marker of inflammation and cancer.^[^ ^249^ ^]^ This nanostructure accurately differentiated inflamed tissue from normal tissue, while its mesoporous silica shell allowed for targeted drug delivery. It quantitatively monitored H₂O₂ production in inflammation, tumors, and osteoarthritis models in rabbits, while tracking anti‐inflammatory treatment efficacy. The integrated SERS and PA signals offer a reliable strategy for multi‐scale, real‐time disease diagnosis and therapeutic monitoring.

Recently, a novel SERS‐based nanoprobe was designed for targeted cancer imaging using DNA origami templates (Figure 12c).^[^ ^221^ ^]^ The nanoprobe consisted of a AuNR dimer assembled by a DNA origami template, functionalized with a DNA aptamer sequence specific to metastatic prostate cancer DU145 cells. It utilized a Raman reporter with vibrational frequencies in the cell‐silent region, enhancing the contrast between the nanoprobe and intrinsic cellular signals. This system demonstrated exceptional specificity, yielding a five‐fold stronger signal in DU145 cells compared to the less metastatic LNCaP cells. The use of DNA origami imparts high spatial addressability, facilitating the fabrication of plasmonic nanostructures with robust SERS signal generation. This multifunctional nanoprobe demonstrates great potential for targeted cancer imaging, offering a scalable, cost‐effective, and reproducible platform for functional SERS nanoprobes. It highlights the versatility of DNA origami in constructing complex nanostructures suitable for large‐scale production, paving the way for advanced applications in cancer research, targeted therapies, and clinical diagnostics.

## 7 DNA‐Assembled Photonic Nanomaterials for Therapy Applications

DNA‐directed photonic nanomaterials, leveraging the versatility of DNA nanotechnology, offer innovative solutions for cancer therapy and the treatment of different diseases. Their programmability provides precise control over size, structure, and functionality, enabling targeted delivery, efficient cellular uptake, and stimuli‐responsive drug release for enhanced therapeutic precision. These constructs excel in photothermal therapy (PTT) by converting NIR into localized heat to selectively destroy cancer cells, and in photodynamic therapy (PDT) by generating reactive oxygen species (ROS) to induce localized cell death. Additionally, their ability to co‐deliver chemotherapeutic drugs and nucleic acids enables synergistic effects that boost treatment efficacy. The drug‐release responses to stimuli such as light further minimize adverse effects on healthy tissues. With modular designs that integrate imaging agents, these photonic systems combine therapy and diagnostics into a unified platform, allowing real‐time monitoring and personalized treatment strategies.

### 7.1 Photothermal Therapy

DNA‐enabled photonic nanoassemblies have advanced PTT by harnessing the programmable assembly of light‐absorbing nanostructures. Upon laser irradiation, they can convert light into localized heat, selectively targeting cancer cells while sparing healthy tissue. Their inherent biocompatibility ensures efficient cellular uptake, minimizing side effects and improving therapeutic outcomes.

**Figure 13:**
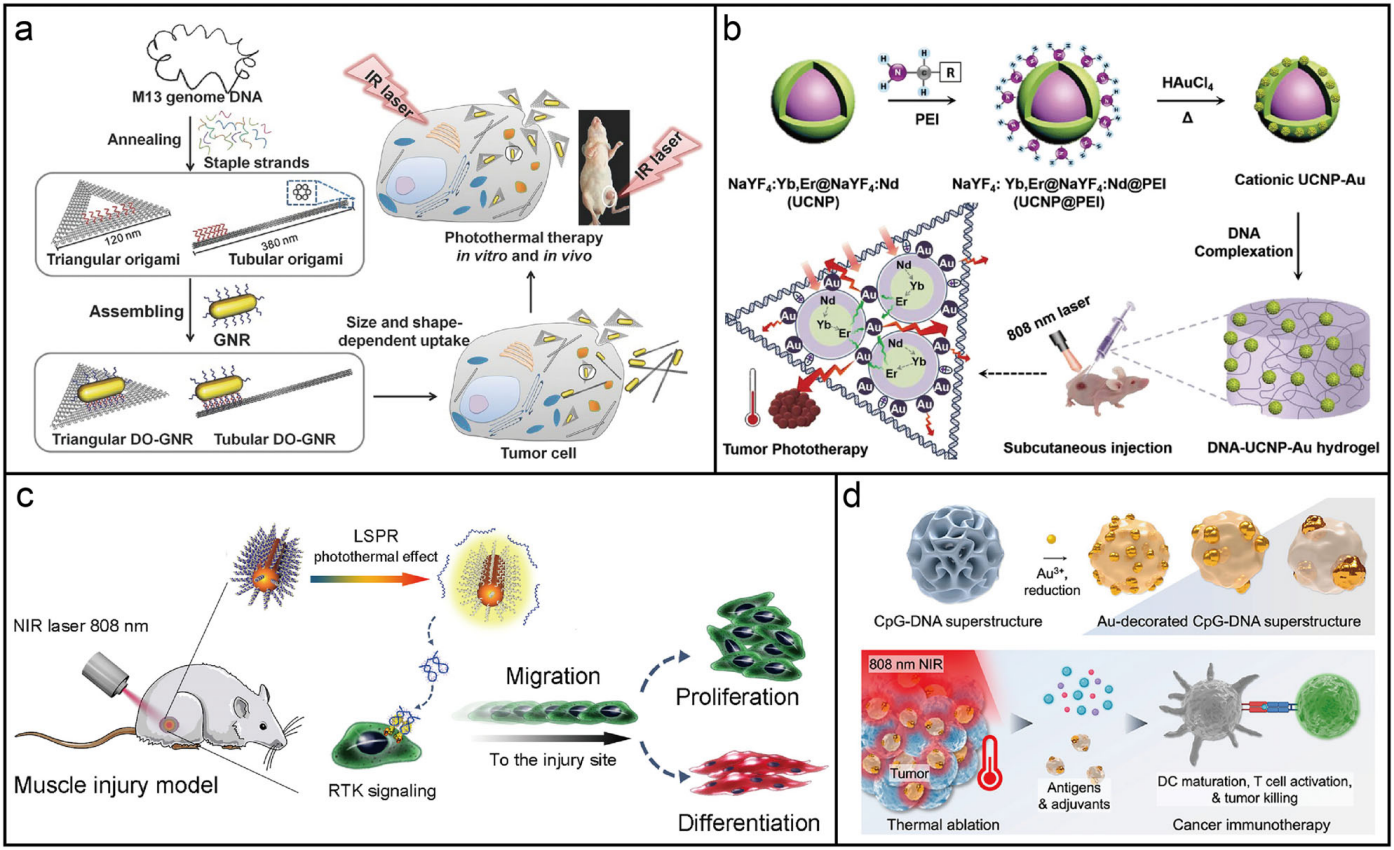
Photothermal therapy using DNA‐directed photonic nanomaterials. a) Self‐assembled DNA origami‐AuNR complex for PTT, demonstrating a significant enhancement in cancer cell uptake.^[^ ^250^ ^]^ Copyright 2015, Wiley. b) Injectable DNA‐inorganic hybrid hydrogels for PTT, with UCNP‐AuNPs enabling NIR‐induced tumor eradication.^[^ ^251^ ^]^ Copyright 2020, Wiley. c) NIR‐activated DNA agonist nanodevice for photothermal control of cell signaling and tissue regeneration via AuNRs.^[^ ^254^ ^]^ Copyright 2019, American Chemical Society. d) DNA superstructure‐templated gold nanocluster formation for photoimmunotherapy, combining photothermal effects and immunostimulants for cancer treatment.^[^ ^255^ ^]^ Copyright 2024, American Chemical Society.

As shown in **Figure** **13**a, a DNA origami‐AuNR complex was developed as a photothermal theranostic platform.^[^ ^250^ ^]^ Upon incubation with MCF‐7 breast cancer cells, the complex showed significantly enhanced cellular uptake compared to bare AuNPs. The triangular‐shaped complex exhibited optimal cellular accumulation and enhanced photothermolysis under two‐photon or NIR laser irradiation. In vivo studies conducted on breast tumor xenografts in mice revealed superior antitumor efficacy for the complex compared to AuNRs alone. These results emphasize the promising capabilities of DNA origami‐guided photonic complexes in cancer diagnosis and treatment, applicable to both in vitro and in vivo settings. To address the challenge of tumor recurrence and improve the limited therapeutic effects of current NIR‐light‐induced PTT, a more effective treatment strategy was developed using a NIR‐light‐responsive injectable DNA‐enabled upconversion and AuNPs hybrid hydrogel (DNA–UCNP‐Au) (Figure 13b).^[^ ^251^ ^]^ As a result of the interaction between DNA strands and UCNP‐Au nanoparticles, the hydrogel exhibited an exceptionally strong photothermal response, reaching a photothermal efficiency of 42.7%, which outperforms that of the original inorganic particles. Peritumoral injection of this hydrogel, followed by 808 nm laser irradiation, effectively eradicated tumors without recurrence, while no adverse effects were observed in normal tissues. Recently, a theoretical study investigated the thermoplasmonic properties of DNA origami‐enabled core/shell toroids for PTT. The study optimized key geometrical parameters and analyzed the effects of metal coatings and random rotations on thermoplasmonic performance to identify configurations with enhanced PTT efficacy and deeper tissue penetration, offering valuable insights for the design of next‐generation DNA‐directed PTT systems.^[^ ^252^ ^]^

In addition to direct treatment through photothermal effects, DNA‐assembled nanoparticle complexes have been utilized for photothermal imaging and precise labeling of cancer cells.^[^ ^253^ ^]^ These constructs offer high spatiotemporal resolution for studying cellular processes and targeting specific tissues, making them valuable for both research and clinical applications. A novel NIR light‐responsive DNA agonist nanodevice was designed to enable non‐genetic modulation of cell signaling and phenotype, offering precise spatiotemporal control over receptor‐mediated cellular behaviors in deep tissues. As illustrated in Figure 13c, the DNA agonist is conjugated to AuNRs. When exposed to NIR light, the photothermal effect of AuNRs, driven by LSPR, induces the release and activation of the DNA agonist. The released agonist then induces the dimerization of receptor tyrosine kinases (RTKs) on the cell surface, initiating downstream signal transduction. This mechanism regulates key cellular processes, including cytoskeletal remodeling, cell polarization, and migration, enabling precise control over receptor‐mediated behaviors in targeted cells.^[^ ^254^ ^]^

To explore the potential of using combinatory therapeutic agents, a multipurpose DNA superstructure was fabricated that combined the photothermal effect of AuNPs with the sequence‐specific immunostimulatory properties of DNA. These constructs functioned as efficient photoimmunotherapy agents.^[^ ^255^ ^]^ As shown in Figure 13d, gold nanoclusters were formed on DNA superstructures through localized reduction, providing control over their mechanical and optical properties. These DNA‐AuNP superstructures exhibited significant photothermal effects, effectively inducing apoptosis in cancer cells. Incorporating immunostimulatory DNA sequences, such as CpG, into these constructs provides a synergistic therapeutic approach to cancer treatment and metastasis inhibition, showing great promise for biomedical applications.

### 7.2 Photodynamic Therapy

The precise structural control, programmability, and biocompatibility of DNA‐directed photonic nanomaterials have enabled the development of advanced photosensitizers for PDT. By leveraging DNA self‐assembly, chiral shell‐satellite nanostructures with remarkable enantiomer‐selective properties have been constructed.^[^ ^256^ ^]^ These nanostructures demonstrated strong chiroptical activity and significantly enhanced ROS generation under circularly polarized light. They achieved quantum yields of up to 1.09, outperforming conventional PDT agents. The enantiomer‐dependent ROS generation further enhanced PDT efficiency, achieving complete tumor ablation both in vitro and in vivo with minimal off‐target cytotoxicity. In addition to their phototherapeutic efficacy, these shell‐satellite nanostructures facilitated multimodal imaging, allowing precise tumor diagnosis and monitoring.

To address the challenges of precise tumor targeting and efficient PDT, a pre‐protective strategy using a DNA/upconversion nanocomposite was developed.^[^ ^257^ ^]^ The nanocomposite consisted of polyacrylic acid ‐coated UCNPs functionalized with folic acid (FA) and chlorin e6 (Ce6), which were attached to DNA sequences of varying lengths. In normal tissues, the longer DNA sequences concealed the FA groups on the shorter DNA, thereby inhibiting their interaction with folate receptors (FRs) on healthy cells. Upon entering the acidic tumor microenvironment, the longer DNA sequences formed C‐quadruplex structures, exposing the FA groups and allowing precise targeting of cancer cells via FA‐FR interactions. Simultaneously, the structural transformation brought the photosensitizer Ce6 into proximity with the UCNPs, enabling FRET to activate Ce6 under NIR light. This process generated singlet oxygen, effectively destroying tumor cells. In vivo studies have demonstrated enhanced tumor accumulation, precise targeting, and significant inhibition of tumor growth using this dynamic nanocomposite. The advantages of DNA in this system include its ability to program responsive structures, adapt to the tumor microenvironment, and provide biocompatibility, all of which are crucial for optimizing PDT efficacy. This approach offers a promising platform for advancing cancer therapy.

### 7.3 Light‐Triggered Drug Delivery

Various DNA‐based light‐responsive strategies, such as light‐induced cargo release, container opening, and targeted activation, have been explored for drug delivery.^[^ ^258^, ^259^, ^260^, ^261^ ^]^ These approaches leverage the programmability of DNA to construct nanostructures that respond to specific light stimuli, enabling spatial and temporal control over drug release while minimizing off‐target effects. The inherent biocompatibility of DNA further enhances their clinical potential. Among these strategies, light‐responsive DNA nanostructures offer unique advantages by utilizing specific light wavelengths to achieve precise drug release, enhancing targeting efficiency while ensuring clinical safety. Their programmability makes them highly effective for applications in diagnostics and therapy.

For example, simple dsDNA‐coated AuNPs functioned as a NIR light‐triggered drug delivery system. Specifically, thiolated dsDNA was conjugated onto AuNPs to host two FDA‐approved breast cancer drugs: docetaxel and lapatinib. The release of docetaxel from the nanoshell‐DNA host complex, triggered by continuous wave laser irradiation, led to enhanced cell death, accompanied by additional nonspecific cell death caused by photothermal heating.^[^ ^262^ ^]^

**Figure 14:**
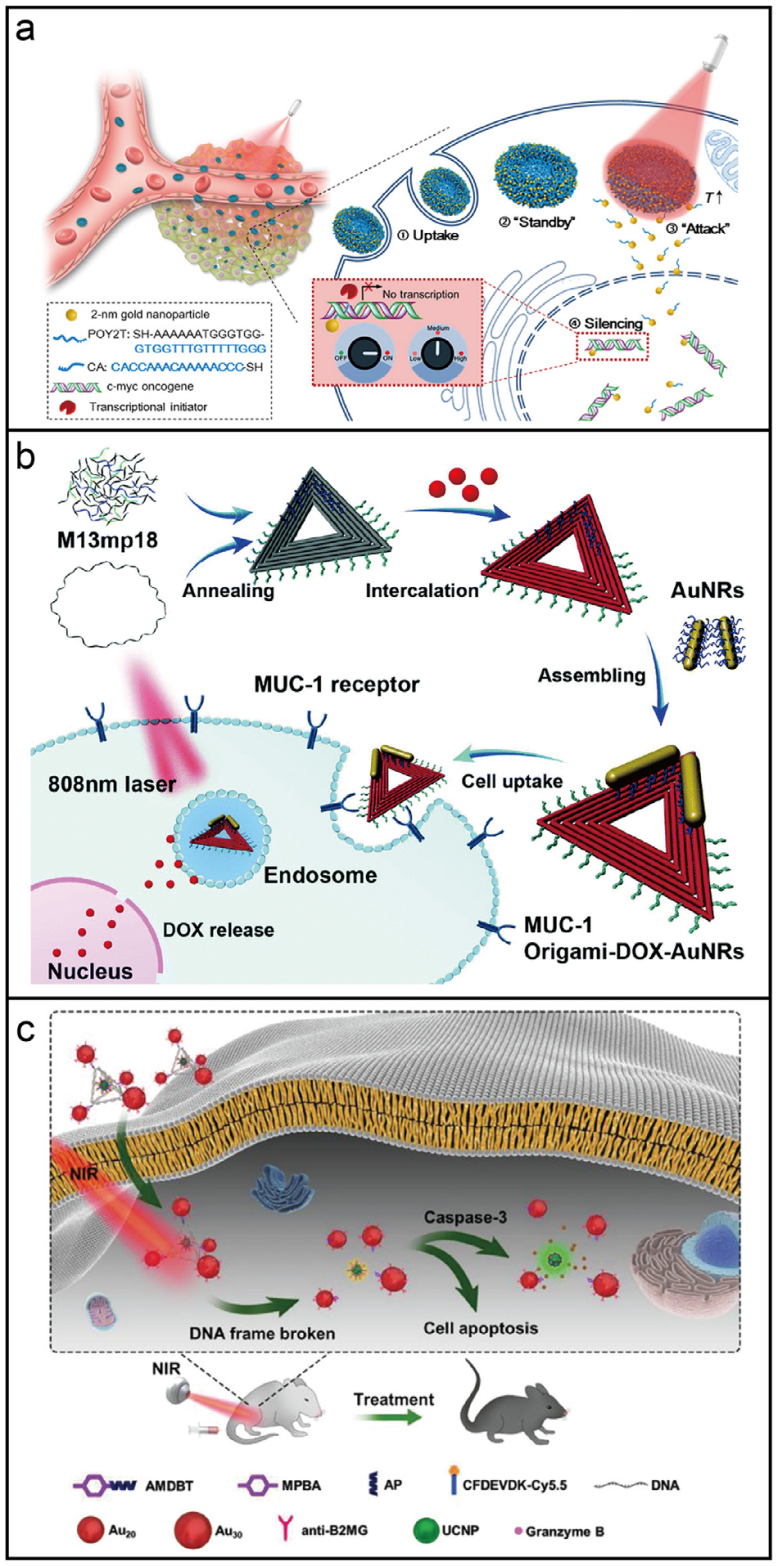
Light‐triggered drug delivery systems. a) Sunflower‐like nanostructures formed by triplex‐forming oligonucleotides and AuNPs for NIR‐triggered delivery of ultrasmall nanoparticles contaning a *c‐myc* oncogene silencing sequence.^[^ ^263^ ^]^ Copyright 2019, AAAS. b) Multifunctional DNA origami carrier for targeted delivery, with DOX intercalated into the DNA structure and AuNRs assembled at specific sites.^[^ ^264^ ^]^ Copyright 2017, Royal Society of Chemistry. c) NIR‐responsive tetrahedron nanostructure composed of UCNPs and 20–30 nm AuNPs for targeted delivery of Granzyme B to senescent cells.^[^ ^265^ ^]^ Copyright 2020, Wiley.

Unlike single nanoparticle‐based delivery systems, the use of DNA‐assembled nanostructures, such as ultrasmall AuNPs, enables precise disassembly and controlled release of therapeutic molecules in response to NIR light. As illustrated in **Figure** **14**a, sunflower‐like nanostructures demonstrated significant NIR absorption and photothermal conversion capabilities.^[^ ^263^ ^]^ Upon NIR irradiation, these structures disassembled, releasing ultrasmall nanoparticles modified with therapeutic sequences (e.g., *c‐myc* oncogene silencing sequences). By regulating the preincubation time and irradiation timing, precise modulation of gene silencing efficacy was achieved, thereby enabling controlled tumor inhibition. This strategy protects therapeutic agents from degradation, enhances specificity, and ensures efficient clinical applications.

DNA origami serves as a multifunctional platform due to its high spatial addressability, allowing integration of therapeutic agents to combat drug resistance in cancer cells. As shown in Figure 14b, DNA origami was used to precisely arrange DOX, AuNRs, and a tumor‐specific aptamer, MUC‐1.^[^ ^264^ ^]^ The aptamer facilitated active targeting, enhancing the internalization within resistant breast cancer cells, MCF‐7. Upon NIR laser irradiation, AuNRs caused hyperthermia, which downregulated the expression of P‐glycoprotein, a multidrug resistance pump, thereby overcoming drug resistance. This approach not only enables controlled release of DOX via DNA‐directed intercalation, but also enhances photothermal ablation, offering a promising approach for addressing drug resistance in cancer therapy.

Besides DNA origami, DNA tetrahedrons formed by DNA hybridization also offer significant advantages for drug delivery, especially in targeting and treating senescent cells. As shown in Figure 14c, a DNA tetrahedron integrates 30 and 20 nm AuNPs, as well as UCNPs conjugated to Granzyme B, for precise drug release.^[^ ^265^ ^]^ Anti‐beta‐2‐microglobulin antibodies attached to the AuNPs allow selective recognition and targeting of senescent cells. When exposed to NIR light, the boronic ester linkage within the tetrahedron was disrupted, releasing Granzyme B directly into senescent cells to induce apoptosis. This approach avoids damage to healthy cells, offering precise control over drug delivery, while enhancing therapeutic efficacy and safety.

### 7.4 Combination Therapy

While each therapeutic modality demonstrates efficacy, they often face limitations. For instance, chemotherapy and PTT are often hindered by multidrug resistance. Although small interfering RNAs (siRNAs) can target and suppress resistance genes, the efficient delivery of these agents alongside chemo‐photothermal agents in vivo remains a significant challenge. Combination therapy integrates multiple therapeutic modalities to enhance efficacy and reduce side effects. The convergence of DNA nanotechnology and photonic nanomaterials enables the development of multifunctional nanosystems for simultaneous drug delivery, PTT, PDT, or chemotherapy. By harnessing the programmability and photonic properties of these systems, combination therapy achieves precision, adaptability, and synergy in addressing complex disease mechanisms and overcoming the limitations of single‐modality treatments.

**Figure 15:**
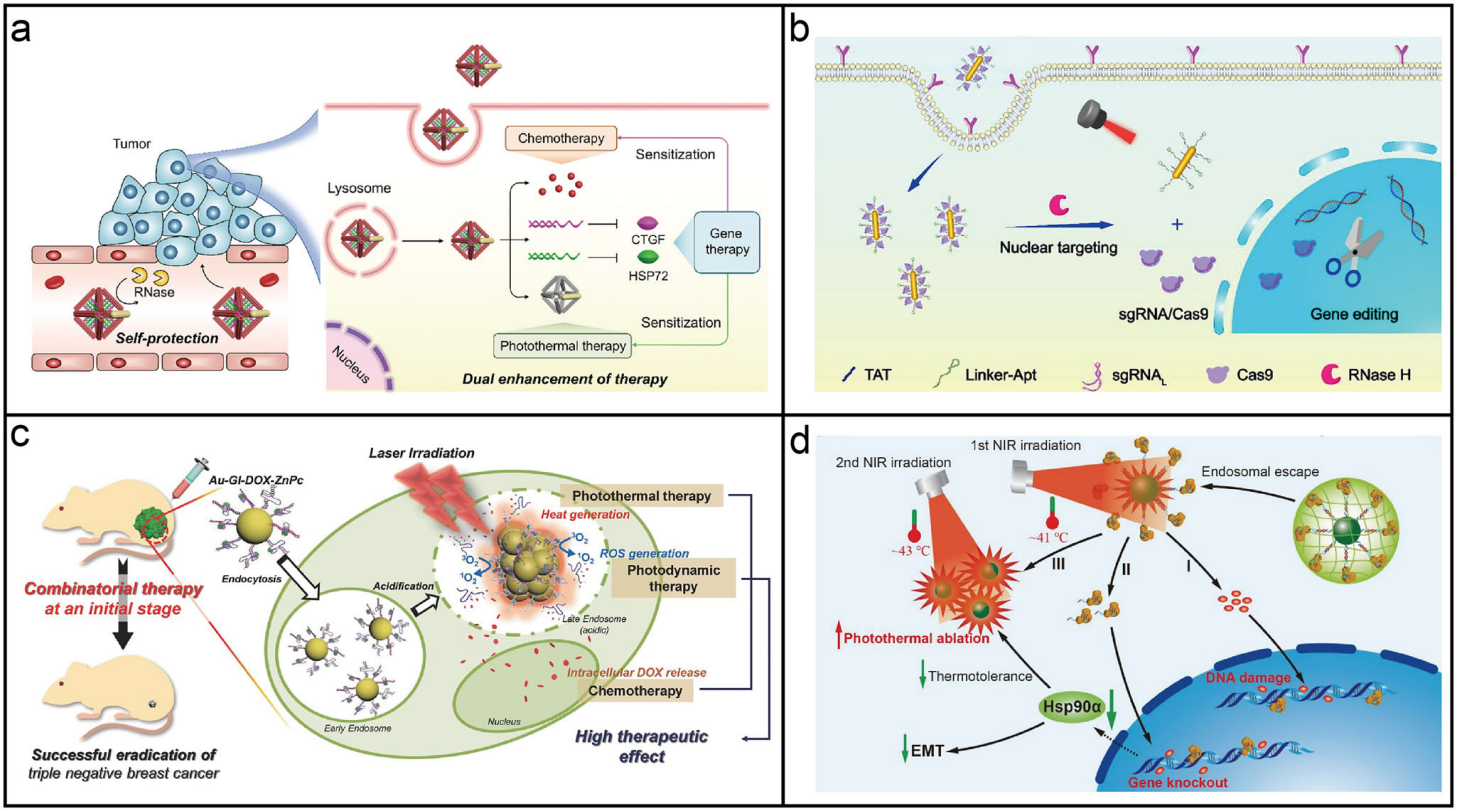
Combination therapy using multifunctional photonic nanomaterials. a) Octahedral DNA origami nanoscaffold designed for siRNA and chemo‐photothermal agent delivery, enabling synergistic cancer therapy through controlled siRNA release and dual enhancement of chemotherapy and PTT.^[^ ^266^ ^]^ Copyright 2021, Wiley. b) Nanoplatform combining sgRNA/Cas9 with a nuclear targeting peptide (TAT) and DNA linker‐functionalized AuNRs for targeted gene editing and efficient tumor inhibition through photothermal treatment.^[^ ^269^ ^]^ Copyright 2021, American Chemical Society. c) Functional DNA‐decorated dynamic gold nanomachine for triple combinatorial anti‐cancer therapy, utilizing pH‐sensitive duplex dissociation for drug release, G‐quadruplex‐assisted photosensitizer delivery, and photothermal ablation upon infrared irradiation.^[^ ^270^ ^]^ Copyright 2027, Wiley. d) NIR‐responsive photothermal nanotherapeutic system designed for the delivery of Cas9 ribonucleoprotein and DOX, facilitating chemotherapy, gene editing, and enhanced mild‐PTT.^[^ ^271^ ^]^ Copyright 2021, Wiley.

As illustrated in **Figure** **15**a, octahedral DNA origami structures were designed as nanocarriers to enable the precise assembly and coordinated delivery of siRNAs, chemotherapeutic agents (such as DOX), and photothermal substances (like AuNRs) for combination cancer therapy.^[^ ^266^ ^]^ These DNA frameworks effectively addressed the challenge of co‐delivering siRNA and chemo‐photothermal agents in vivo. The rigid inner cavity of these DNA frameworks protected siRNAs from degradation in circulation, allowing for dual sensitization of cancer cells to both chemo‐drugs and hyperthermia. This approach enabled the simultaneous suppression of resistance‐related genes, such as connective tissue growth factor (CTGF) and heat shock protein 72 (HSP72), enhancing the therapeutic response. This system provides a synergistic effect that maximizes therapeutic efficacy and minimizes systemic toxicity, demonstrating the potential for precisely targeted medication in cancer therapy. Furthermore, synergistic photothermal‐chemotherapy for cancer has also been developed using pH‐responsive DNA‐conjugated AuNPs,^[^ ^267^ ^]^ and the coupling of DNA‐templated Ag nanoclusters with polydopamine nanoparticles.^[^ ^268^ ^]^

The CRISPR‐Cas9 gene‐editing system shows significant potential for tumor treatment due to its powerful ability to edit oncogenes. However, a key challenge remains in efficiently delivering the sgRNA‐Cas9 complex to target tumor cells. By employing a thiolated DNA linker, the sgRNA‐Cas9 complex can be effectively attached to AuNRs through hybridization between the 3′ overhang of sgRNA and the DNA linker. Building on this, a novel co‐assembled nanoplatform was developed, where AuNRs serve as the scaffold, functionalized with a custom DNA linker, a cell‐specific aptamer, and a nuclear‐targeting peptide. (Figure 15b).^[^ ^269^ ^]^ This nanoplatform facilitated targeted gene editing by effectively delivering the sgRNA‐Cas9 complex through RNA‐DNA hybridization. Additionally, the system also incorporated mild photothermal treatment using AuNRs, enhancing the inhibition of tumor cell proliferation. This combined strategy demonstrates the potential of integrating gene editing with photothermal therapy for enhanced therapeutic efficacy in cancer treatment.

Beyond dual‐therapy approaches, triple combinatorial anti‐tumor therapies have also been introduced. As shown in Figure 15c, a DNA‐AuNP nanomachine equipped with i‐Motif and G‐Quadruplex was engineered for cancer treatment, utilizing the combinatorial effects of chemotherapy, PDT, and PTT.^[^ ^270^ ^]^ This nanomachine capitalized on the unique optical properties of AuNPs, particularly their size‐dependent response to NIR light. A cytosine‐rich i‐motif sequence enabled pH‐sensitive duplex dissociation and aggregation, facilitating controlled anticancer drug release and photothermal ablation under NIR irradiation. Additionally, the G‐quadruplex structure ensured stable loading and delivery of photosensitizers, enabling effective PDT through red light illumination. The G‐quadruplex assisted in generating reactive oxygen species, pH‐responsive dynamic aggregation, and subsequent drug release. The therapeutic efficacy of this multifunctional nanoplatform was validated both in vitro and in vivo using a triple‐negative breast cancer model, highlighting its capacity for synergistic cancer therapy.

In another example, a multifunctional nanotherapeutic platform based on copper sulfide (CuS) was developed, responsive to NIR light and temperature changes. This platform enables the controlled release of CRISPR‐Cas9 ribonucleoprotein (RNP) and DOX, facilitating a synergistic combination therapy that integrates mild PTT, chemotherapy, and gene therapy. (Figure 15d).^[^ ^271^ ^]^ The CuS semiconductor functioned as a “photothermal converter,” effectively transforming NIR light (808 nm) into localized heat for photothermal stimulation. The double‐stranded structure formed between CuS nanoparticle‐conjugated DNA fragments and single‐guide RNA (sgRNA) acted as a regulated component in response to this stimulation, enabling accurate gene editing and drug delivery. By targeting heat shock protein 90 (Hsp90α) with Cas9 RNP, the tumor's thermal tolerance was reduced, thereby enhancing the mild‐PTT effects (≈43 °C). The platform's efficacy was demonstrated through two NIR light irradiations in both in vitro and in vivo settings, showing significant synergistic therapeutic effects compared to PTT alone. This externally regulated approach provides a flexible strategy for precise gene editing and targeted drug release, optimizing therapeutic outcomes while reducing adverse effects.

### 7.5 Integrated Diagnosis and Therapy

Integrated diagnosis and therapy, or theranostics, combines diagnostic and therapeutic functions within a single platform, revolutionizing precision medicine. DNA‐directed assembly of photonic nanomaterials uniquely facilitates this integration by leveraging the programmability, specificity, and versatility of DNA. The inherent ability of DNA to encode precise structural and functional information enables the controlled spatial arrangement of photonic nanomaterials, optimizing their optical properties for real‐time imaging. Simultaneously, DNA serves as a highly specific targeting agent and carrier for therapeutic payloads, ensuring efficient delivery to diseased sites. This dual role enhances the synergy between diagnosis and therapy, allowing for precise disease targeting, real‐time monitoring, and adaptive treatment.^[^ ^272^ ^]^

**Figure 16:**
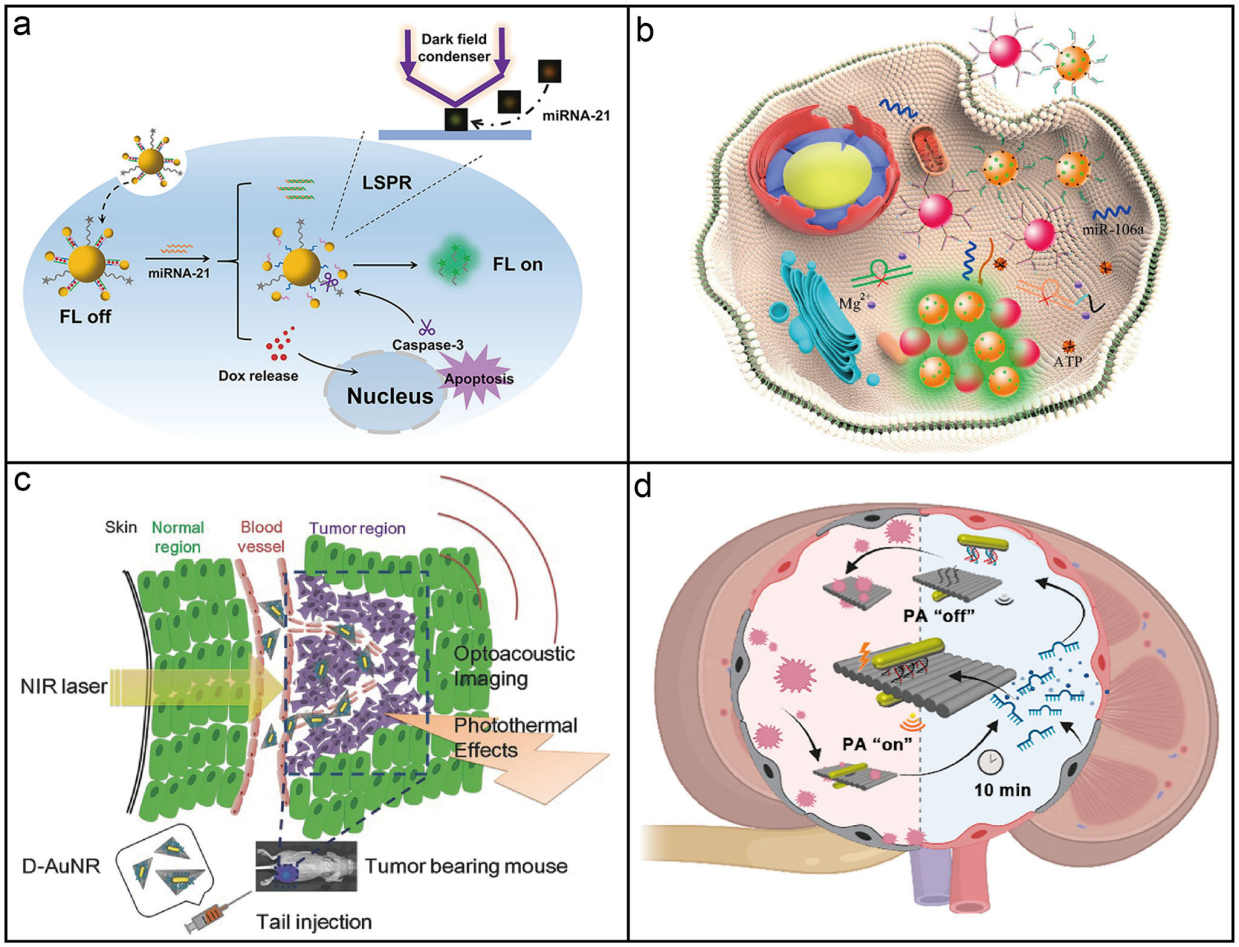
Multifunctional photonic nanomaterials for simultaneous diagnosis and therapy. a) Multifunctional plasmonic core‐satellite nanoprobe for targeted drug release, miRNA detection, and therapy evaluation.^[^ ^244^ ^]^ Copyright 2021, American Chemical Society. b) Theranostic nanosystem based on AuNPs that combines cascaded SERS imaging with miRNA‐responsive DNAzyme‐mediated gene silencing therapy for precise cancer diagnosis and treatment.^[^ ^247^ ^]^ Copyright 2022, American Chemical Society. c) DNA origami‐AuNR hybrid nanoprobe system showing AuNRs assembled on a triangular DNA origami structure for simultaneous targeted imaging and photothermal therapy.^[^ ^273^ ^]^ Copyright 2016, Wiley. d) MicroRNA‐responsive AuNR dimer templated by a DNA origami nanostructure for early acute kidney injury diagnosis and treatment.^[^ ^275^ ^]^ Copyright 2022, American Chemical Society.

LSPRs combined with DFM offer excellent sensitivity and real‐time monitoring capabilities, enabling precise biosensing and bioimaging, while integrating targeted drug delivery and chemotherapy into a single multifunctional platform. As shown in **Figure** **16**a, a DOX‐loaded plasmonic core‐satellite nanoprobe combined microRNA detection, targeted drug release, and therapy evaluation in a single platform.^[^ ^244^ ^]^ Triggered by miRNA‐21, the nanostructure disassembled, generating LSPR signals with visible color changes and spectral shifts for ultrasensitive miRNA‐21 detection and tumor cell identification. Concurrently, DOX release induced apoptosis and activated caspase‐3, allowing fluorescent monitoring of therapeutic effects. This multifunctional nanoprobe integrates molecular imaging, therapeutic evaluation, and chemotherapy, addressing key challenges in cancer theranostics and advancing precision medicine.

SERS provides a unique molecular signature spectrum with good photostability, allowing ultrasensitive and specific detection of biomolecules in complex biological environments. Building on these advantages, a novel AuNP‐based theranostic nanosystem was developed for precise cancer diagnosis and treatment (Figure 16b).^[^ ^247^ ^]^ The system employed two types of AuNPs: one modified with Y‐motifs and the other functionalized with Raman molecules and dsDNA linkers. When intracellular cancer‐associated miR‐106a is detected, the Y‐motifs and dsDNA linkers undergo conformational shifts driven by miRNA and ATP, resulting in the release of miRNA for further amplification. This process initiates the formation of AuNP network nanostructures, creating numerous SERS hotspots that enable highly sensitive and specific detection of cancer cells. At the same time, the activation of DNAzyme facilitates the Mg^2^⁺‐dependent cleavage of Survivin and c‐Jun mRNAs, effectively silencing both genes for a dual therapeutic effect.

PA imaging is a molecular imaging technique that combines optical and acoustic components, enabling deep tissue penetration with high spatial resolution and contrast. By utilizing NIR light, several DNA‐directed photonic systems that integrate PA imaging with various therapeutic strategies have been developed to allow real‐time functional imaging across a wide range of biomedical applications.^[^ ^256^, ^273^, ^274^, ^275^ ^]^ For instance, as demonstrated in Figure 16c, a PA imaging agent was developed by self‐assembling AuNRs onto DNA origami nanostructures.^[^ ^273^ ^]^ This hybrid system combined the advantages of AuNRs and DNA origami, serving as an effective probe and contrast agent in PA imaging with improved imaging quality and reduced dosage. It also reacted to NIR light for photothermal therapy, significantly suppressing tumor recurrence and extending the survival of affected mice. A microRNA‐responsive nanoantenna, composed of two AuNRs connected by a rectangular DNA origami framework, was designed to harness the ROS scavenging and local oxidative stress‐reducing properties of DNA origami. This innovative system enables early detection and targeted treatment of acute kidney injury (AKI) (Figure 16d). The nanoantenna's LSPR peak was within the NIR‐II window (∼1060 nm), allowing for PA imaging in deep tissues. In AKI mice, the nanoantenna showed exclusive kidney retention and reduced PA signals due to the detachment of AuNRs from DNA origami in response to over‐expressed miR‐21. This provided a LOD of 2.8 nm for early diagnosis, approximately two orders of magnitude earlier than other probes. The naked origami scaffold also scavenged more ROS, helping to alleviate ischemic AKI. Furthermore, integrating PA imaging with other imaging modalities, such as a dual model with Raman imaging^[^ ^85^ ^]^ and a triple model with fluorescence and SERS imaging,^[^ ^276^ ^]^ can enhance the accuracy and versatility of DNA‐directed photonic materials for simultaneous diagnosis and therapy.

Additionally, while DNA‐directed assemblies have garnered significant interest in therapeutics and diagnostics, their stability in both intracellular and extracellular environments is challenged by low cation concentrations and nuclease activity under physiological conditions.^[^ ^277^ ^]^ To address this, various stabilization strategies, particularly for DNA origami, have been developed, including protective coatings,^[^ ^278^ ^]^ covalent structural modifications,^[^ ^279^ ^]^ protective encapsulation,^[^ ^280^ ^]^ backbone modifications,^[^ ^281^ ^]^ and environmental adjustments.^[^ ^282^ ^]^ The selection of an optimal protection method should consider structure‐dependent stability, functional accessibility, and potential modifications. Furthermore, thorough verification is essential to ensure compatibility with engineered sites and intended applications, particularly in targeted delivery.

## 8 Summary and Outlook

The DNA‐directed assembly of photonic nanomaterials has emerged as a transformative approach for creating highly organized and multifunctional platforms, offering immense potential across optical and biomedical applications.^[^ ^26^ ^]^ By leveraging the programmability, specificity, and versatility of DNA, researchers have unlocked unprecedented control over the spatial arrangement of photonic components, enabling precise modulation of light‐matter interactions.^[^ ^40^ ^]^ These advancements have driven innovations in diagnostics and therapy, addressing some of the most complex challenges in photonics and nanomedicine.

Despite the exciting potential, several challenges remain to fully realize the promise of this technology. One pressing challenge is scalability. Although DNA‐assembled nanostructures provide unmatched precision, their assembly methods remain labor‐intensive and costly, limiting their practicality for clinical and industrial deployment.^[^ ^15^ ^]^ To overcome this limitation, the development of automated, high‐throughput fabrication techniques, such as microfluidics, or robotic systems, will be essential. Another significant challenge is the inherent instability of DNA in physiological environments. To address this, strategies such as chemical modifications, functionalization with biocompatible polymers, or integration with protective nanomaterials can enhance structural robustness, ensuring reliable performance in biological systems.^[^ ^277^ ^]^ Additionally, minimizing immunogenicity is critical. This can be achieved by carefully selecting materials and implementing surface modifications that preserve the structural integrity and functionality of the nanomaterials.^[^ ^283^ ^]^ Furthermore, addressing the safety concerns associated with the biocompatibility of metal nanoparticles is vital. Ongoing research is focused on enhancing their safety profiles, with strategies such as the incorporation of biocompatible ligands playing a key role in improving their suitability for diagnostic and therapeutic applications.^[^ ^4^ ^]^

From a photonic standpoint, DNA‐directed assemblies represent an exciting frontier in the bottom‐up fabrication of optical systems. The ability to precisely position nanoparticles, fluorophores, and other optical components enables the creation of advanced photonic architectures that are unattainable through traditional lithographic techniques. Taking advantage of photonic effects, such as enhanced fluorescence and SERS, DNA nanotechnology has expanded the design space for photonic systems. Moving forward, exploiting the second near‐infrared (NIR‐II) window (≈1000–1700 nm) holds immense promise for applications requiring deep tissue penetration, reduced scattering, and high imaging resolution, particularly in biomedical imaging and photothermal therapies.^[^ ^284^ ^]^ Additionally, hybrid architectures that combine DNA nanotechnology with emerging photonic materials, such as van der Waals materials and perovskites, could drive the discovery of novel light‐matter interactions and functionalities.^[^ ^285^ ^]^

The integration of artificial intelligence (AI) and computational photonics provides a promising avenue for accelerating the development of DNA‐directed photonic systems.^[^ ^286^ ^]^ AI‐driven models can optimize the design of nanostructures, predict their optical properties, and guide their assembly, while computational approaches can model complex light‐matter interactions with high accuracy. These tools will not only improve efficiency but also enable the creation of adaptive and stimuli‐responsive photonic systems that can dynamically adjust their optical properties in response to environmental cues such as light, temperature, or biomolecules. Such innovations could revolutionize real‐time diagnostics and smart therapeutic interventions.

The interdisciplinary nature of DNA‐directed photonic systems underscores their transformative potential. By bridging molecular biology, materials science, and optics, these assemblies not only advance precision medicine but also set the stage for addressing broader challenges in biomedical innovation. The programmability of DNA, coupled with its ability to organize optical components at the nanoscale, exemplifies the synergy between biology and photonics in designing next‐generation technologies. Dynamic and reconfigurable DNA‐based photonic architectures, in particular, represent a paradigm shift, enabling systems that are both multifunctional and adaptive to their environment. In conclusion, the DNA‐directed assembly of photonic nanomaterials represents a powerful platform for creating versatile and functional systems with applications spanning biosensing, bioimaging, diagnostics, and therapy. As researchers continue to refine their designs and integrate emerging technologies, these systems are poised to redefine the landscape of nanotechnology and photonics, unlocking transformative opportunities across science and healthcare.

## Conflict of Interest

The authors declare no conflict of interest.
